# Unexpected palaeodiversity of omaliine rove beetles in Eocene Baltic amber (Coleoptera, Staphylinidae, Omaliinae)

**DOI:** 10.3897/zookeys.863.34662

**Published:** 2019-07-11

**Authors:** Alexey V. Shavrin, Shûhei Yamamoto

**Affiliations:** 1 Institute of Life Sciences and Technologies, Daugavpils University, Vienibas 13, Daugavpils, LV-5401, Latvia Daugavpils University Daugavpils Latvia; 2 Integrative Research Center, Field Museum of Natural History, 1400 S Lake Shore Drive, Chicago, IL 60605-2496, USA Field Museum of Natural History Chicago United States of America

**Keywords:** Anthophagini, Eusphalerini, Omaliini, fossil, micro-CT

## Abstract

Fossil records of the subfamily Omaliinae are fragmentary and most of them are less informative compression fossils. Baltic amber from the mid-Eocene of northern Europe is one of the most important sources of insect fossils, but only two reliably placed omaliines have been described. Here, we provide a general overview of this subfamily in Baltic amber. In total, five new extinct species of four genera in three tribes are described and illustrated: *Geodromicusbalticus***sp. nov.** (Anthophagini), *Eusphalerumkanti***sp. nov.** (Eusphalerini), *Paraphloeostibamorosa***sp. nov.**, *Phyllodrepadaedali***sp. nov.**, and *Ph.icari***sp. nov.** (Omaliini). Additionally, we report on four species belonging to *Eusphalerum*, which remain unnamed, from the same amber deposit. The records of *Eusphalerum* include the first fossils of the tribe Eusphalerini, while that of *Geodromicus* may represent the second and the first definitive fossil record of the genus and tribe Anthophagini. Our discoveries highlight the unexpected palaeodiversity of Omaliinae in Baltic amber, further reinforcing the coexistence of thermophilous and temperate-loving beetles in Baltic amber and potentially indicating wetland and riparian habitats of amber-producing forests.

## Introduction

With 1639 species in 117 extant and 14 extinct genera (Thayer 2016; A.F. Newton unpublished database 17 Jan. 2019), the rove beetle subfamily Omaliinae (Staphylinidae) is a relatively large group, currently composed of seven tribes: Anthophagini Thomson, 1859, Aphaenostemmini Peyerimhoff, 1914, Corneolabiini Steel, 1950, Coryphiini Jakobson, 1908, Eusphalerini Hatch, 1957, Hadrognathini Portevin, 1929 and Omaliini W.S. MacLeay, 1825. However, McKenna et al. (2015) recently demonstrated the non-monophyly of Omaliinae with respect to three other subfamilies (i.e., Empelinae Newton & Thayer, 1992, Glypholomatinae Jeannel, 1962, and Microsilphinae Crowson, 1950) in the "Omaliine group of subfamilies based on two molecular markers. Their result supports the earlier results of Thayer (2000) based on larval morphology (although larvae of Empelinae are still unknown and therefore were not included in her study). These four subfamilies combined formed a monophyletic clade, sister to Proteininae (McKenna et al. 2015). Members of the Omaliinae are distributed worldwide, with the greatest diversity in the Holarctic and Oriental regions, predominantly in montane areas. A revision and clear diagnosis of Omaliinae still do not exist, and thus, the status of many supraspecific taxa is still unclear due to the difficulties of formally placing them within tribes (Newton and Thayer 1992, 1995). Omaliinae, or even the Omaliine group, have often been considered to be plesiomorphy-rich among Staphylinidae (Thayer 2016). However, this was not supported by the comprehensive molecular phylogenetic study by McKenna et al. (2015). The presence of paired ocelli in most taxa of Omaliinae has often been regarded as one of the most important characters to define the subfamily. Nevertheless, it is unclear whether ocelli should be interpreted as primitive or even apomorphic (Newton and Thayer 1995; Leschen and Beutel 2004; Cai et al. 2013; Thayer 2016). The extant omaliines are further characterized by having antennae inserted under the lateral margins of the frons, tarsal formula 5-5-5 (except Corneolabiini, 4-4-4; Steel 1950), procoxal cavities opened behind, well-developed prosternal and postprocoxal processes, procoxae conical and prominent, abdomen with six visible sternites, abdominal intersegmental membranes attached apically and with brick-wall-like pattern of sclerites, all spiracles well developed and functional, presence of wing-folding patches of microtrichia on some abdominal tergites, and anterior projection of abdominal sternite VIII with well-developed defensive glands (e.g. Klinger 1980; Dettner and Reissenweber 1991), as well as some features of genitalia and genital segments of both male and female (Thayer 1985; Newton et al. 2000; Peris et al. 2014; Zanetti et al. 2016).

A brief history of fossil Omaliinae was recently provided by Chatzimanolis (2018). Fossil records of omaliines are relatively prevalent. Compared to the high diversity and abundance of extant Omaliinae, however, the records of extinct omaliines are still significantly fragmentary. Many of them are described with short descriptions, incomplete illustrations and problematic systematic placements (Chatzimanolis 2018). Hence, it prevents a comparison of these fossils to each other and to recent taxa. In addition, several extinct genera are known as “tribe *incertae sedis*” and not placed in any of the tribes mainly because of their poor preservation or difficulty in interpreting their morphology (Schaufuss 1890; Tikhomirova 1968; Ryvkin 1985, 1990; Herman 2001). Chatzimanolis (2018) listed five Jurassic omaliine genera (Tikhomirova 1968; Ryvkin 1985): †*Archodromus* Tikhomirova, †*Globoides* Tikhomirova, and †*Porrhodromus* Tikhomirova from the Upper Jurassic of Karatau, Kazakhstan; †*Eophyllodrepa* Ryvkin from the Middle-Upper Jurassic of Novospasskoe, Russia; and †*Morsum* Ryvkin from the Middle Jurassic of Kubekovo, Western Siberia. Two genera †*Daiodromus* Ryvkin and †*Prodaia* Ryvkin are known from the Upper Jurassic of Daya, Russian Transbaikalia, although Chatzimanolis et al. (2012) regarded them as Lower Cretaceous taxa. Later, Cai and Huang (2013) added the extinct genus †*Sinanthobium* Cai & Huang from the Middle Jurassic of Inner Mongolia, China. Compared to Jurassic compressions, only a handful of fossils are known from the Cretaceous, with only a single compression fossil, genus †*Mesodeliphrum* Ryvkin, described from the Lower Cretaceous of Turga, Transbaikalia (Ryvkin 1990). In addition, Peris et al. (2014) recently reported the first Mesozoic amber genus †*Duocalcar* Peris & Thayer (Omaliini) from opaque Lower Cretaceous French (Charentes) amber and visualized the beetle fossil by using phase-contrast Synchrotron Radiation x-ray microtomography (PPC-SR X-ray μCT).

The Cenozoic omaliine fauna is also far from well understood. Scudder (1900) described the compression fossil species *Geodromicusabditus* Scudder (Anthophagini), from the Upper Eocene of Florissant, USA, although the preservation is not adequate to assess its systematic position. For example, the whole head was lost prior to description (Scudder 1900), which makes its generic identification highly doubtful or impossible. Nevertheless, according to the original description, the general body shape of *G.abditus* is similar to that of *Geodromicus* or some other related genera (for example *Microedus*), but there is no information about structure of the head and mouthparts. Another fossil species, *Omaliumantiquorum* Wickham (Omaliini), with *Proteinus*-shaped body (Wickham 1913: pl. 5 fig. 3), is described from the same horizon (Wickham 1913), but again, the preservation of this fossil is insufficient to justify its taxonomic placement. Although the description is too short for final conclusions (Wickham 1913), this taxon may not belong to *Omalium* due to the proportions of the markedly transverse pronotum and wide elytra, which are consistent with many *Proteinus* (Proteininae) species. Other pre-Quaternary records include *Anthophagusgiebeli* Heyden & Heyden (Anthophagini) from the Oligocene of Germany (Heyden and Heyden 1866), *Omaliumprotogaeae* Heer from the Miocene of Croatia (Heer 1847) and some unnamed fossils (e.g. Hopkins et al. 1971; Archibald and Mathewes 2000; Kiselev and Nazarov 2009). All these compression fossils need re-examination as generic assignments by the early paleontologists are doubtful. For example, *A.giebeli* can be assigned neither to *Anthophagus* nor Anthophagini. Based on the small body with two darkened spots in middle of the pronotum, it may belong to *Eusphalerum* or a small Omaliini (*Phloeonomus*-like) considering the shape of the very wide abdomen (Heyden and Heyden 1866). Schaufuss (1890) described *Pseudolesteuainsinuans* Schaufuss from the mid-Eocene Baltic amber (ca 44 Ma) but this fossil may not even belong to Omaliinae (Zanetti et al. 2016). Unfortunately, Schaufuss’s material was likely to be lost or scattered during World War II (Vitali 2006); thus, it is probably impossible to re-examine the type specimen. The only definitive omaliines in Baltic amber were recently described by Zanetti et al. (2016), representing two Omaliini species: *Paraphloeostibaelectrica* Zanetti et al. and *Phyllodrepaantiqua* Zanetti et al. They were visualized with the PPC-SR X-ray μCT method, illuminating fine morphological details. Further, Hieke and Pietrzeniuk (1984) noted an “*Anthobium*” from Baltic amber identified by E. Reitter; however, this species has not been formally described (see also the history of confusion between *Anthobium*, *Lathrimaeum* and *Eusphalerum* in Tottenham (1939) and Shavrin and Smetana (2017)).

The present paper provides an overview of the remarkable and unexplored palaeofauna of Omaliinae in Baltic amber. We report at least 18 fossil beetles in seven amber pieces, with the descriptions of five new extinct species in four extant genera from the tribes Anthophagini, Eusphalerini and Omaliini. The new species and unnamed specimens of *Eusphalerum* Kraatz represent the first definitive fossil of Eusphalerini, while that of *Geodromicus* Redtenbacher may represent the second and the first definitive fossil record of the genus and Anthophagini. These discoveries are significant for future phylogenetic and paleontological studies of the subfamily Omaliinae and related taxa.

## Materials and methods

Eighteen adults in seven Baltic amber pieces were used in our study. Nearly all studied material is deposited in the Gantz Family Collections Center, Field Museum of Natural History (FMNH), Chicago, USA, with the assigned specimen numbers from FMNHINS-3260628 to FMNHINS-3260632 with addition of FMNHINS-3965993, but a single amber piece is derived from the private collection of V.I. Alekseev (Kaliningrad, Russia), under the registration number AWI-045. Each piece of amber is placed in a small transparent rectangular plastic envelope with the labels within. The age of Baltic amber is of great debate, with estimates from the Lower Eocene to Lower Oligocene (e.g. Perkovsky et al. 2007; Weitschat and Wichard 2010; Alekseev 2013; Bogri et al. 2018), although it is generally accepted as middle to upper Eocene. Here we tentatively follow the mid-Eocene (Lutetian: 44.1 ± 1.1 Ma) age based on the most recent estimations obtained by the absolute dating analyses of glauconites from Sambia Peninsula (Wappler 2005). The staphylinid fauna in Baltic amber is diverse and abundant, with the following 12 extant subfamilies recorded: Aleocharinae, Euaesthetinae, Omaliinae, Oxyporinae, Paederinae, Piestinae, Proteininae, Pselaphinae, Scydmaeninae, Staphylininae, Steninae, and Tachyporinae (e.g. Chatzimanolis and Engel 2011; Alekseev 2013; Cai et al. 2017; Yamamoto and Maruyama 2017). The amber pieces originate from the Baltic Sea Coast: Yantarny, Kaliningrad, Russia (FMNHINS-3965993, FMNHINS-3260629, FMNHINS-3260632, AWI-045); Wisła River, Gdańsk, Poland (FMNHINS-3260628); and the Baltic Sea Coast without further information (FMNHINS-3260630). The second author (SY) further prepared two specimens (FMNHINS-3260628 and FMNHINS-3260629) by polishing with emery papers of different grain sizes and a plastic buffing cloth.

The following measurements are used in this paper and abbreviated as follows:

HW maximum width of head including eyes;

HL length of head (from base of labrum to neck constriction along head midline in dorsal view or from apical margin of mentum to neck constriction in ventral view (*G.balticus* sp. nov.));

OL ocular length (longitudinal);

PLL×PLW (II, III) length×width of segments II and III of labial palpi;

PML×PMW (III, IV) length×width of segments III and IV of maxillary palpi;

AL length of antenna;

PL length of pronotum;

PW maximum width of pronotum;

ESL sutural length of elytra (length of elytra from the apex of scutellum to the posterior margin of sutural angle);

EW maximum width of elytra together;

MTbL length of metatibia;

MTrL length of metatarsus;

AW maximum width of abdomen (at segment IV);

TL total length (from anterior margin of clypeus to apex of abdomen).

All measurements are given in millimeters and were made with a stereoscopic microscope equipped with an ocular micrometer. Some measurements of the body were difficult to do because of the specimen's partial visibility and orientation within the amber pieces; the resulting approximate values are marked with “~”, and the cases when measurements were not possible are marked with “?”. The description of the preservation of the material is given below the type material listing in a separate paragraph. The type labels are cited in inverted commas and separated from each other by a comma, different lines in labels of the types and historic labels are separated with ‘|’; explanations of the type labels are given in square brackets, necessary notes within the label are given in angle brackets.

Specimens were examined using Nikon SMZ 745T and Nikon Eclipse E200 stereomicroscopes. A digital camera (Sony Alpha DSLR-A300) was used for photographs of habitus of *Geodromicusbalticus* sp. nov. Other photographs were produced using a Canon 80D digital camera with a Canon MP-E 65 mm macro lens (F2.8, 1–5×), equipped with a Canon MT-24EX macro twin lite flash as light source. Then, image stacks were carried out using CombineZM software (Alan Hadley, Sheffield, UK). All figures were modified using Adobe Photoshop software. For one paratype (FMNHINS-3260630) of *Eusphalerumkanti* sp. nov., images were generated using x-ray micro-computed tomography (μ-CT), acquired with a micro-focus x-ray CT system (inspeXio SMX-100CT; Shimadzu) through the courtesy of Shimadzu Corp. (Kyoto, Japan). It was scanned at 60 kV under 60 μA, resulting in a voxel size of 5.0 μm. Specific settings of the scan are confidential and retained by the company. Rendering of the image volume was carried out using VGstudio max v. 2.2 (Volume Graphics, Heidelberg, Germany).

## Systematic Palaeontology

### Order Coleoptera Linnaeus, 1758

#### Family Staphylinidae Latreille, 1802

##### Subfamily Omaliinae MacLeay, 1825

**Systematic placement of fossils.** The characters of the subfamily by which the fossil specimens describe here are unambiguously referred to Omaliinae are: shape of the body is variable but in general more or less wide, with short and less flexible abdomen than most staphylinids; elytra are variable in length and sometimes distinctly elongate and covering the entire abdomen (Newton and Thayer 1995); dorsal surface of the head, more or less close to hind margin, usually with ocelli (e.g. Hatch 1957; Moore and Legner 1979; Newton et al. 2000; Leschen and Beutel 2004; Thayer 2016), reduced in some taxa (see below); apical maxillary palpomere as wide as penultimate segment, but in Coryphiini and some taxa of Anthophagini and Omaliini it can be distinctly narrower; antennae attached under lateral margins of frons, filiform, moniliform or clavate; coxal cavities usually open; postcoxal process well developed (with some exceptions; see Newton et al. 2000); procoxae conical and prominent; epistomal suture absent, posterior face of metacoxa vertical (Newton and Thayer 1995; Thayer 2016); tarsal formula 5-5-5 (4-4-4 in Corneolabiini); abdomen with six visible sternites; abdominal tergites three to seven, usually with one pair of paratergites; intersegmental membranes attached apically and with brick-wall structures (e.g. Hammond 1971).

###### Tribe Anthophagini Thomson, 1859

####### Genus *Geodromicus* Redtenbacher, 1857

**Type species.***Staphylinusplagiatus* Fabricius, 1798

######## † Geodromicus balticus

Taxon classificationAnimaliaColeopteraStaphylinidae

Shavrin & Yamamoto sp. nov.

http://zoobank.org/16755333-4CBD-4485-B51A-6B64459253B3

**Figures 1–16.**
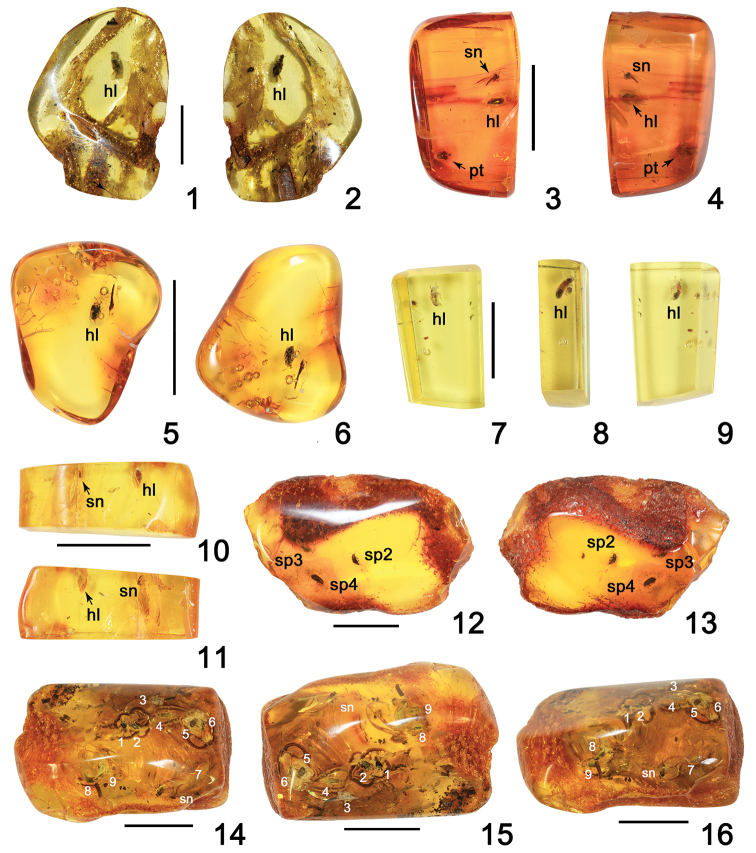
Amber specimens with inclusions of Omaliinae: **1, 2***Geodromicusbalticus* sp. nov. **3, 4***Eusphalerumkanti* sp. nov. **5, 6***Paraphloeostibamorosa* sp. nov. **7–9***Phyllodrepadaedali* sp. nov. **10, 11***Ph.icari* sp. nov. **12, 13***Eusphalerum* sp. 2 (sp2), *Eu.* sp. 3 (sp3) and *Eu.* sp.4 (sp4) **14–16***Eu.* sp. 1 (specimens 1 to 9 (in the text: no. 1 to no. 9). Abbreviations: hl = holotype, pt = paratype, sn = syninclusion. Scale bars: 1.0 cm (**1–6, 10–16**), 0.5 cm (**7–9**).

**Figures 17, 18.**
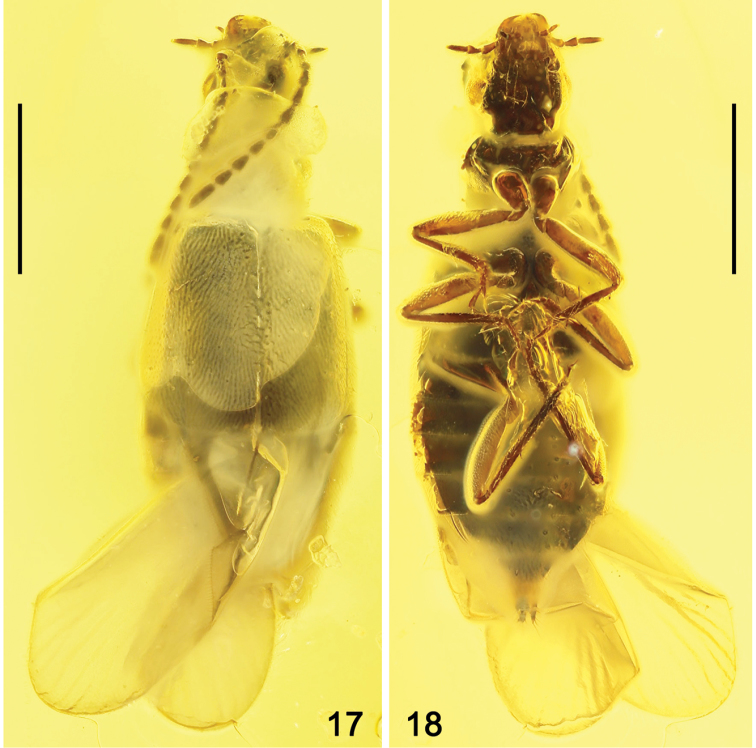
Habitus photographs of *Geodromicusbalticus* sp. nov. **17** Dorsal view **18** Ventral view. Scale bars: 1.0 mm.

**Figures 19, 20.**
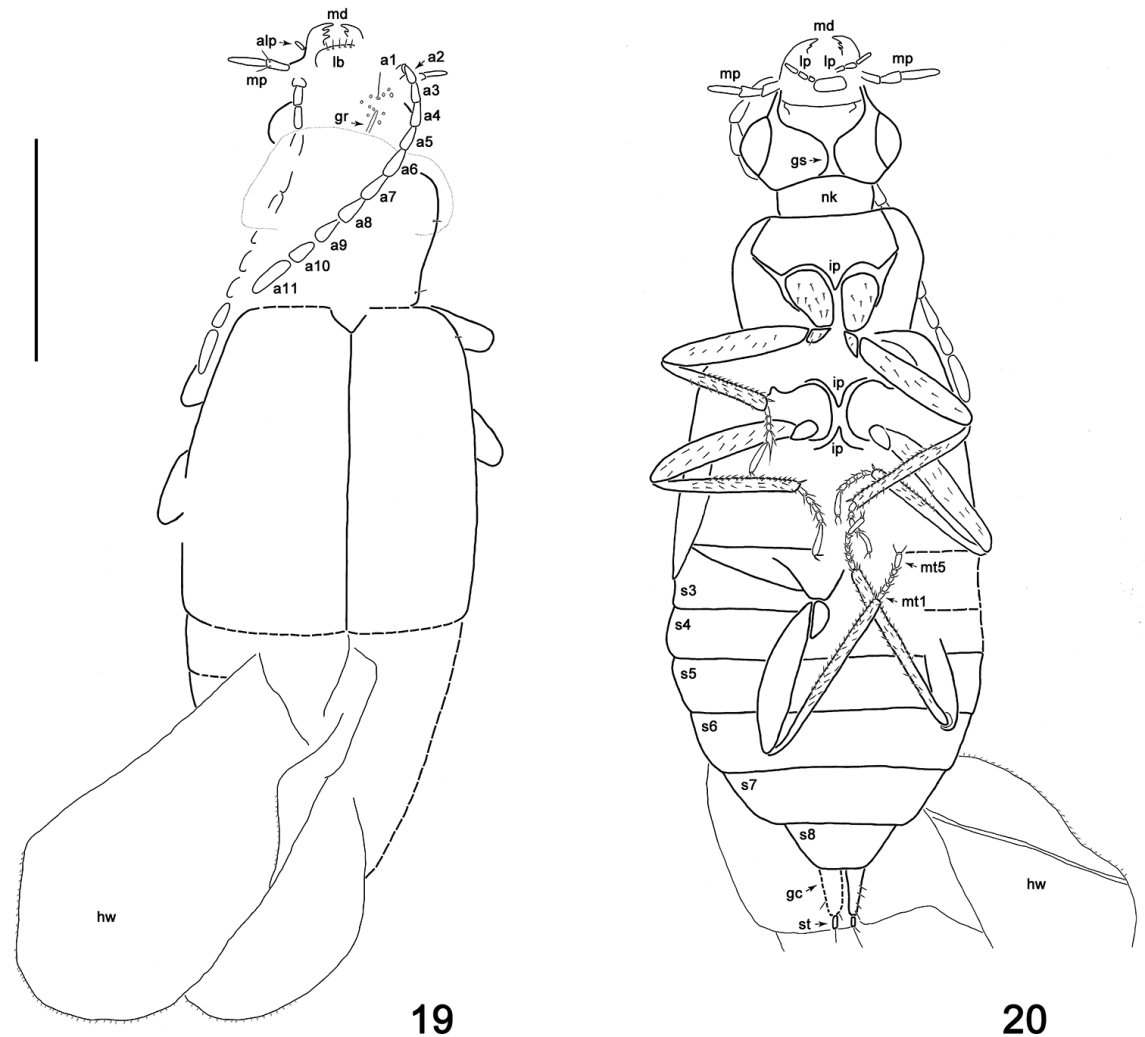
Habitus drawings of *Geodromicusbalticus* sp. nov. **19** Dorsal view **20** ventral view. Abbreviations: a1–a11 = antennomeres 1–11; alp = apical labial palpomere; gc = gonocoxite; gr = groove; gs = gular suture; hw = hind wing; ip = intercoxal process; lb = labrum; lp = labial palpi; md = mandibles; mp = maxillar palpus; mt1, mt5 = metatarsomeres; nk = neck; s3–s8 = sternites 3–8; st = stylus. Scale bar: 1.0 mm.

Figures 1 , 2 , 17–18 , 19–20

######### Type materials examined.

Holotype: female, FMNHINS-3965993, complete specimen as inclusion in a piece of light yellow Baltic amber, 3.4 cm × 2.4 cm × 0.5 cm in size (Figs 1, 2), with glued small paper on plastic envelope labeled “6083”, with three colour photographs of habitus of the beetle (two of dorsal and one of ventral view) with rectangular stamp on the back of each labeled “Certificate 6083 [handwritten in blue] | Natural Baltic Amber with Inclusions | expert Jonas Damzen | International Amber Association | Names of Inclusions: | Staphylinidae [handwritten in blue] | Rove beetle [handwritten in blue]” <with additional round stamp on the left side: “+SOCIETAS SVCCINORVM+INTERNATIONALIS”]>, with the following labels: “Baltic amber | Yantarny, Kaliningrad | Russia | (S. Yamamoto Coll.) | ?Geodromicus | Omaliinae, Anthophagini | Protobiae with minute | hairs | tarsi with long hairs” <rectangular label; handwritten on both sides of the label>, “HOLOTYPE | *Geodromicus* | *balticus* sp. nov. | Shavrin A. & Yamamoto S. des. 2019” <red rectangular label, printed> (FMNH).

######### Preservation.

The specimen is poorly visible because it is partially covered with white cloud of microbubbles created by decay products interacting with resin, a characteristic of authentic Baltic amber (Cai and Huang 2013). This is especially noticeable on the anterior half of the body, under the apical and basal portions of the head, including the usual location of ocelli, and most of the pronotum. The abdominal tergites are not visible dorsally, as they are covered by the hind wings. The ventral side of the specimen is visible in detail except for the basal portion of the thoracic sclerites.

######### Locality and horizon.

Baltic amber from Yantarny, Kaliningrad, westernmost Russia; mid-Eocene (ca 44 Ma; Wappler 2005).

######### Description.

Measurements: HW (ventral): 0.76; HL (ventral): ~0.40; OL (ventral): 0.25; PLL×PLW (II, III): II: 0.05 × 0.03, III: 0.08 × 0.02; PML × PMW (III, IV): III: 0.10 × 0.06, IV: 0.16 × 0.05; PL (ventral): ~0.47; PW (ventral): ~0.87; ESL: 1.40; EW: 1.51; MTbL: 1.00; MTrL: 0.36 (I–IV: 0.20; V: 0.16); AW (IV): 1.41; TL: 3.80 (head of specimen slightly out of pronotum, thus the total length likely to be slightly shorter). Antennomeres with lengths × widths: 1: ? × 0.07; 2: 0.16 × 0.06; 3: 0.11 × 0.06; 4–5: 0.15 × 0.05; 6–7: 0.15 × 0.07; 8: 0.14 × 0.07; 9–10: 0.12 × 0.07; 11: 0.25 × 0.07.

Body elongate; forebody convex. Specimen dark-brown and glossy, with antennomeres brown, mouthparts reddish-brown, legs yellow-brown with a somewhat darkened tibia. Habitus as in Figures 17–20.

Head transverse, slightly elevated in middle, about twice as wide as long, with short temples, moderately strongly narrowing toward neck, with diagonal moderately deep grooves (visible only apical part of left groove), reaching level of apical third of eye; gular sutures slightly separated at narrowest point on level of basal third of length of eyes (Fig. 20). Eyes large and widely convex, with medium-sized facets. Medioapical portion with irregular, dense and moderately deep, small punctation, without microsculpture, basal portion of head between eyes and gular sutures with dense diagonal mesh. Middle portion of swollen neck (ventral view) with dense isodiametric microsculpture (Fig. 18). Labrum transverse, with moderately widely rounded apical margin and elongate apical setae, without visible median emargination. Mandibles with strongly curved acute apex; left mandible with two elongate subapical teeth on inner side of cutting edge; distal third of cutting edge of right mandible not clearly visible, with elongate tooth somewhat shorter than that in left mandible. Maxillary palpi moderately long, with several long setae on apical margins of palpomeres 2 and 3; palpomere 2 narrower basally, gradually and slightly widened apicad; palpomere 3 distinctly longer than broad, narrow basally, markedly widened apicad; apical palpomere elongate, 1.8 times as long as penultimate segment one and visibly narrower at base than apex of penultimate one, somewhat parallel-sided in middle, slightly narrowed toward moderately acute apex (Figs 17–20). Labial palpomeres 2 and 3 distinctly longer than their width, apical palpomere 1.6 times as long as preceding segment, gradually narrowing toward apex from middle. Antenna reaching basal third length of elytra, with moderately wide antennomeres, with short dense setation; antennomere 2 slightly narrower than 1; antennomere 3 slightly shorter than antennomere 2, narrow basally and slightly widened apicad; antennomeres 4 and 5 three times as long as broad; antennomeres 6 and 7 slightly longer and distinctly wider than antennomere 5; antennomere 8 twice as wide as long; antennomeres 9 and 10 slightly shorter than antennomere 8; apical antennomere elongate, twice as long as penultimate segment and more than three times as long as broad (Figs 17, 19).

Pronotum transverse, about 1.3 times as wide as long, slightly wider than head, widest slightly in front of middle, markedly more narrowed posterad than anterad, indistinctly emarginate laterally; anterior angles widely rounded, posterior angles obtuse. Lateral portions of pronotum with small irregular punctation, without microsculpture. Pubescence appears regular, accumbent. Pronotal hypomeron and postcoxal process well developed; intercoxal process almost reaching basal third of length of procoxae, with acute sharp apex; pronotosternal suture distinct; mesoventrite with acute intercoxal process, reaching basal third of mesocoxae; metaventrite broad, with moderately acute apex of intercoxal process, not reaching mesosternal process (Figs 18, 20). Median part of prosternum with very sparse, irregular, small punctation; metaventrite with dense small punctation (Fig. 18); prosternal process with dense isodiametric microsculpture.

Elytra slightly broader than long, reaching apical margin of abdominal tergite III, markedly more than twice as long as pronotum, gradually widened apicad, with straight hind margin (Figs 17, 19). Punctation dense, small and deep, markedly smaller in basal portion, near scutellum and along suture. Pubescence regular, accumbent. Hind wings fully developed (Figs 17, 18).

Legs of moderately similar length, slender and moderately long; procoxae wide, protruding ventrad; mesocoxae large and oval, contiguous; metacoxae strongly transverse; protrochanter narrow, elongate; mesotrochanter relatively small, semioval; metatrochanter elongate; femora widest about middle; pro- and mesotibiae about as long as femora, slightly widened from narrowest basal portions toward middle, covered with regular moderately short pubescence and elongated setae on lateral margins (more visible in protibiae); metatibia markedly longer than metafemora and more than twice as long as metatarsus; apical metatarsomere slightly shorter than preceding tarsomeres together; tarsal claws simple and moderately long, without modifications (Figs 18, 20).

Abdomen slightly narrower than elytra (Figs 17–20). Abdominal tergite III to IV similar in width, beginning from segment V gradually narrowed apicad; tergite VII strongly narrowed to truncate apex (Figs 18, 20).

Male unknown.

Female. Apical margin of abdominal sternite VIII straight (Figs 18, 20). Genital segment with elongate gonocoxites, and moderately small, narrow styli (Fig. 20).

######### Etymology.

The specific epithet is the Latinized adjective derived from the name of the Baltic Sea.

######### Remarks.

Based on the shape of elongate antennomeres 8–10, the general shape of the apical maxillary palpus with elongate apical palpomere not dramatically narrower than the penultimate one, and on the length of tarsomeres 1–4 together distinctly longer than apical tarsomere, the studied specimen undoubtedly belongs to the tribe Anthophagini (Moore and Legner 1979; Newton and Thayer 1995; Newton et al. 2000). Judging from the combination of visible morphological details of the fossil, such as shapes of the forebody, maxillary palpomeres, gonocoxites, and mandibles with developed large teeth on inner side of the cutting edge, as well as by presence of distinct grooves in front of ocelli, the species belongs to the *Hygrogeus* complex of genera (Zerche 1992, 2003). The representatives of these taxa are widespread in the Holarctic Region and contain several genera reaching their greatest diversity in Central and East Asia (Shavrin 2017a). Unfortunately, the condition of the specimen described here does not allow the observation of the presence of neither the anteocellar impressions nor the ocelli. The presence of impression between ocelli depends on the degree of convexity of head and can be significantly variable among genera and even among species of a species group, from indistinct to very deep. Regarding ocelli, these structures can be large and very convex, small, flattened, and indistinct, or reduced (sometimes in one genus) as in the tribe Anthophagini, but also in Coryphiini, Eusphalerini, and Omaliini (Zerche 1990, 1992; Shavrin 2016). Besides proportions of the forebody, internal and external structure of the aedeagus, genera of the *Hygrogeus* complex can vary by the shape of the apical two maxillary palpomeres (Coiffait 1981; Zerche 1992). The fossil cannot be member of *Altaiodromicus* Zerche, 1992, *Hygrodromicus* Tronquet, 1981, *Liophilydrodes* Nakane, 1983, *Microedus* LeConte, 1874, or *Philydrodes* Bernhauer, 1929, because members of these taxa share a short and very narrow apical maxillary palpomere. The new species cannot be placed in *Trichodromeus* Luze, 1903, species of which share a moderately small apical conical-shaped maxillary palpomere, shorter than an indistinctly widened apicad penultimate segment, or *Paratrichodromeus* Zerche, 1992, species of which have an apical maxillary palpomere distinctly narrower and shorter than the preceding segment. Additionally, from all these taxa the new species differs by the following: from *Altaiodromicus*, *Hygrodromicus*, and *Trichodromeus* by the larger eyes, less transverse head and pronotum, and elongate elytra gradually widened apicad; from *Liophilydrodes* and *Microedus* by longer elytra and absence of microsculpture between punctures on the head; from *Philydrodes* by the smaller head, larger pronotum, wider and longer elytra; from *Paratrichodromeus* by the wider pronotum and elytra and shorter antennae. The elongate apical and penultimate segment of the maxillary palpomere in this fossil are somewhat like that of some Asian species of the genus *Hygrogeus* Mulsant & Rey, 1880, especially the narrowed apical portion of the apical segment as in European *H.aemulus* (Rosenhauer, 1847). In general, the fossil differs from *Hygrogeus* by the slightly convex body, distinctly larger eyes and shorter temples, significantly shorter labial palpomeres and markedly wider pronotum. The relatively small body and its coloration, slightly convex dorsal side of the head, similar location of grooves and shape of mandibles are like some species of the genus *Anthophagus* Gravenhorst, 1802. However, it differs from *Anthophagus* by the shape of the apical maxillary palpomere (in *Anthophagus* significantly narrowed apicad and shorter than preceding palpomere), wider pronotum with markedly transverse prosternum and distinctly elongate mesosternal process (*Anthophagus* with a small mesosternal process extending short distance between coxae; Moore and Legner 1979), and, more importantly, different shape and structure of metatarsi: the first metatarsomere very short as opposed to markedly elongate as in *Anthophagus* (sometimes slightly shorter than apical tarsomere) and absence of modifications at base of tarsal claws (*Anthophagus* with two membranous patches at the base).

Based on the general shapes of the forebody, eyes, gular sutures, preapical and apical maxillary palpomeres, and antennomeres, as well as characters of the punctation and microsculpture of the body, shapes of thoracic sclerites, and length of basal metatarsomere, the new species can be placed as a putative *Geodromicus*. The extant representatives of the genus are widely distributed in the Holarctic Region, reaching their greatest diversity in Asia. The genus includes more than 120 species, the majority of which are distributed in the eastern Palaearctic Region and strongly associated with mountain regions (Herman 2001; Schülke and Smetana 2015; Shavrin 2018). According to the observed morphological data, it is rather difficult to place the new species into one of the subgenera (*Geodromicus* sensu stricto or *Brachydromicus* A. Bordoni, 1993) or any species group because the subgeneric subdivision seems to be artificial and species group placements were provided only for some taxa of the western (Bordoni 1984) and eastern (Shavrin 2018) Palaearctic and based on external and internal morphology of the aedeagus. The species can be tentatively compared with the smallest specimens (about 4.00 mm length, known to the first author) of the Palaearctic species *G.plagiatus* (Fabricius, 1798).

From all species of the genus, *G.balticus* sp. nov. differs by the markedly elongate apical segment of maxillary palpi. It highlights the need to revise the supraspecific taxonomy of the *Hygrogeus* complex, some of which have unclear status.

###### Tribe Eusphalerini Hatch, 1957

####### Genus *Eusphalerum* Kraatz, 1857

**Type species.***Anthobiumtriviale* Erichson, 1839 (synonym of *Eusphalerumprimulae* Stephens, 1834)

######## † Eusphalerum kanti

Taxon classificationAnimaliaColeopteraStaphylinidae

Shavrin & Yamamoto sp. nov.

http://zoobank.org/7C377BF2-1233-4E7D-AFDB-CDF6E0EDD22B

Figures 3 , 4 , 21–23 , 24–29 , 30–33 , 34–44

######### Type materials examined.

Holotype (male) and paratype (female), FMNHINS-3260630, complete specimens as inclusions in a piece of dark yellow to reddish orange Baltic amber, 21.6 mm × 12.7 mm × 6.3 mm in size (Figs 3, 4), with the following labels: “SYAC 0027 | Baltic | prob. Anthobium” <rectangular small label, handwritten>, “07[printed] 09 [handwritten] | Baltic / Dominican | Larva/Adult (× 2) [printed] [handwritten] | ? Anthobium [handwritten] | Axel Niggeloh | Schalksmuehle” <large rectangular label, printed>, “15[printed]01[handwritten] – SYAC 00[printed] 27[handwritten] | Baltic / Burmite | Other: | Larva / Adult | prob. Anthobium [handwritten] | 2 in amber [handwritten] | Baltic Sea coast [handwritten] | Shûhei Yamamoto’s | Amber Collection” <large rectangular label, printed>, “[FMNH barcode at left side of label] FMNHINS | 3965993 | FIELD MUSEUM | AMBER” <small rectangular label, printed>, “HOLOTYPE | *Eusphalerum* | *kanti* sp. nov. | Shavrin A. & Yamamoto S. des. 2019” <red rectangular label, printed>, “PARATYPE | *Eusphalerum* | *kanti* sp. nov. | Shavrin A. & Yamamoto S. des. 2018” <red rectangular label, printed> (FMNH).

######### Preservation.

The holotype is best observed on its dorsal side, close to the surface of the amber piece and with apical part of the body somewhat deeper (Fig. 3): head, pronotum and basal portion of elytra are visible from the lateral edge of the amber. The paratype is oriented dorsolaterally and located close to the outer surface of the amber piece (Fig. 3). Syninclusion: imago of Diptera about 2.00 mm length, including wings.

######### Locality and horizon.

Baltic amber from Baltic Sea Coast, further details unknown; mid-Eocene (ca 44 Ma; Wappler 2005).

######### Description.

Measurements (*n* = 2): HW (holotype): 0.47; HL (holotype): 0.33; OL (paratype): 0.18; AL (paratype): 0.75; PML × PMW (III, IV): III: 0.05 × 0.02, IV: 0.11 × 0.02; PL (holotype): 0.42; PW: 0.77; ESL (paratype): 1.25; EW (paratype): 1.15; MTbL (paratype): 0.42; MTrL (paratype): 0.24 (I–IV: 0.12; V: 0.12); AW: ?; TL: 2.60 (holotype)–2.70 (paratype). Antennomeres with lengths × widths (paratype): 1: 0.12 × 0.04; 2: 0.06 × 0.02; 3: 0.07 × 0.02; 4: 0.06 × 0.02; 5–6: 0.06 × 0.03; 7: 0.05 × 0.04; 8: 0.05 × 0.05; 9–10: 0.05 × 0.06; 11: 0.12 × 0.06.

Body elongate, convex (Figs 21, 24, 34, 37); body laterally as in Figure 36; body dorsolaterally as in Figure 23; body ventrally as in Figures 22, 25, 35. The specimens appear black, with mouthparts, antennae and legs yellow-brown; tarsi and basal portion of apical maxillary palpomere yellow. Body glossy and glabrous, without visible setation; antennomeres with elongate setae (Fig. 28).

**Figures 21–23.**
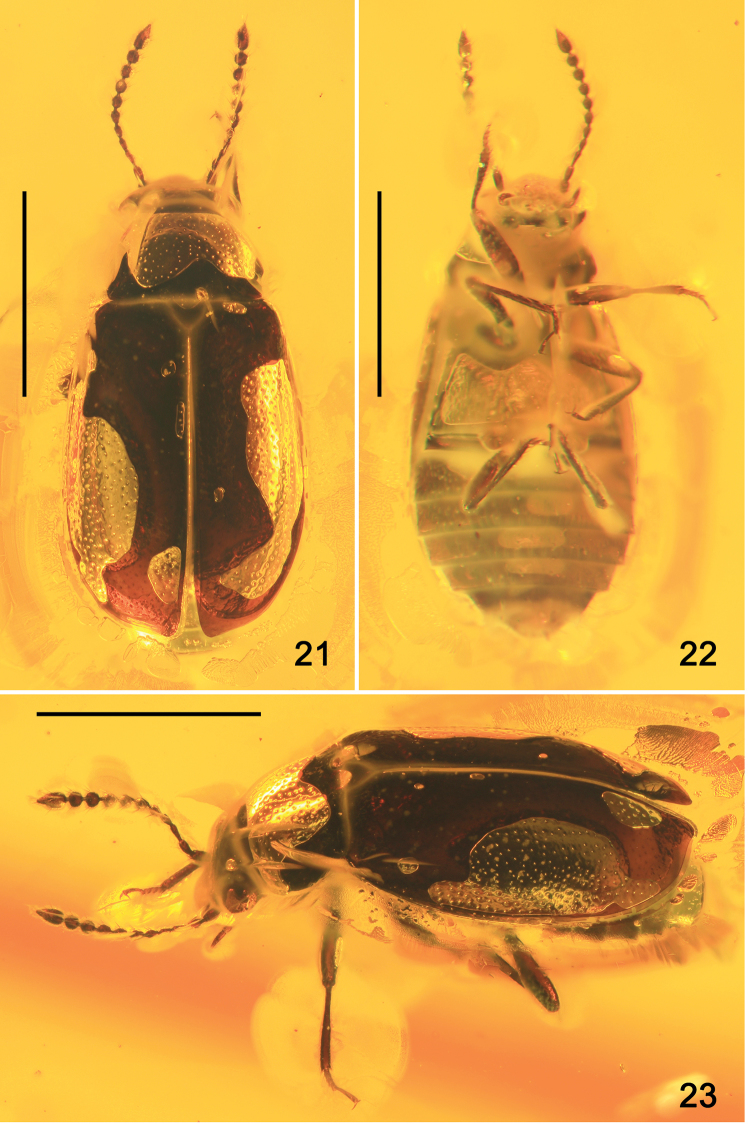
Habitus of *Eusphalerumkanti* sp. nov. (paratype) **21** dorsal view **22** ventral view **23** dorsolateral view. Scale bars: 1.0 mm.

Head about 1.4 times as wide as long (Figs 29, 39); middle portion of head slightly flattened, without visible grooves in front of ocelli, median impressions and occipital line; postocular carina smooth and indistinct. Head laterally as in Figure 42 and dorsolaterally as in Figure 28. Head with moderately irregular, dense and small punctation, markedly denser on posterior portion; middle part of neck with sparse small punctures (Fig. 43); infraorbital ridges with indistinct diagonal small meshes between punctures. Eyes large, widely convex (Figs 35, 37). Ocelli large, situated at level of posterior margins of eyes (Figs 28, 37), distance between ocelli distinctly longer than distance between ocellus and posterior margin of eye. Apical segment of maxillary palpi elongate, twice as long as preceding segment, about same width in middle as preceding segment, from middle gradually narrowed apicad (Figs 22–23, 28). Gular sutures with rounded apical parts, widely separated from each other (Figs 35, 38). Antenna (Figs 21–23, 25, 28, 29) moderately long, slightly exceeding shoulders of elytra, with elongate setae; basal antennomere wide and oblong, antennomere 2 slightly swollen and elongate, antennomere 3 thin and long, antennomere 4 slightly wider than antennomere 3, antennomeres 5 and 6 twice as long as wide, antennomeres 7 and 8 slightly and antennomeres 9 and 10 distinctly transverse, apical antennomere twice as long as wide, strongly narrowed in apical third toward acute apex.

**Figures 24–29.**
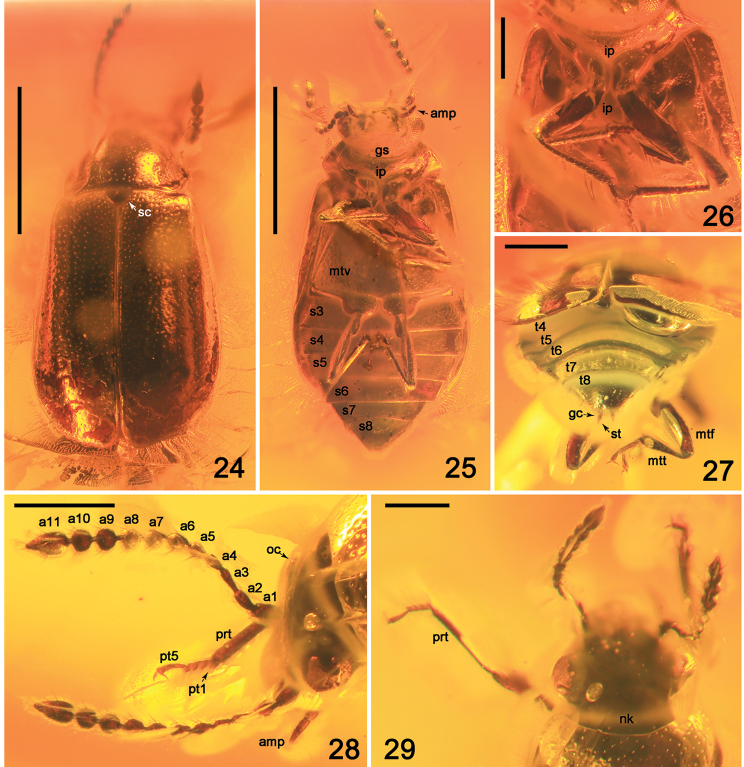
*Eusphalerumkanti* sp. nov. (holotype: **24–26, 29** paratype: **27, 28**) **24** habitus, dorsal view **25** habitus, ventral view **26** thoracic sclerites and legs, ventral view **27** apical part of elytra, abdomen and hind legs, posterodorsal view **28** head and antennae, dorsolateral view **29** head, antennae and forelegs, dorsal view. Abbreviations: a1–a11 = antennomeres; amp = apical maxillary palpomere; gc = gonocoxite; gs = gular suture; ip = intercoxal process; nk = neck; mtf = metafemur; mtt = metatibia; mtv = metaventrite; oc = ocellus; prt = protibia; pt1, pt5 = protarsomeres 1 and 5; s3–s8 = sternites III–VIII; sc = scutellum; st = stylus; t4–t8 = tergites IV–VIII. Scale bars: 1.0 mm (**24, 25**), 0.3 mm (**26–29**).

Pronotum slightly convex, moderately short and transverse, 1.8 times as wide as long, 1.6 times as wide as head, widest at about middle, distinctly more narrowed posterad than anterad (Figs 21, 24, 37); apical margin straight, distinctly narrower than posterior margin; anterior angles widely rounded and distinctly protruded anterad (Figs 39, 43); posterior angles widely rounded; lateral margins distinctly emarginate, without crenulation on lateral edges (Figs 37, 39); pronotum with moderately widely elevated middle portion (Fig. 37), with very indistinct small transverse impression in mediobasal third; lateral portions narrowly but distinctly explanate, each with distinct moderately deep semioval impression at middle (Fig. 39). Pronotum with irregular small punctation like that on head but slightly deeper, markedly sparser in mediobasal and lateral portions; median portion with very indistinct transverse microsculpture. Prosternum with moderately wide intercoxal process (Figs 25, 26, 35, 38). Mesoventrite with thin, elongate and acute intercoxal process indistinctly reaching apical third of mesocoxae (Figs 35, 41). Scutellum large and wide, with several very small punctures in basal portion (Figs 21, 23, 24). Metaventrite convex (Fig. 36), with wide and deep metacoxal cavities and moderately wide metaventral process, reaching middle of mesocoxae, not contacting with apex of mesosternal process (Figs 25, 35, 41). Median part of prosternum and metaventrite with moderately dense small punctation (Figs 25, 35).

**Figures 30–33.**
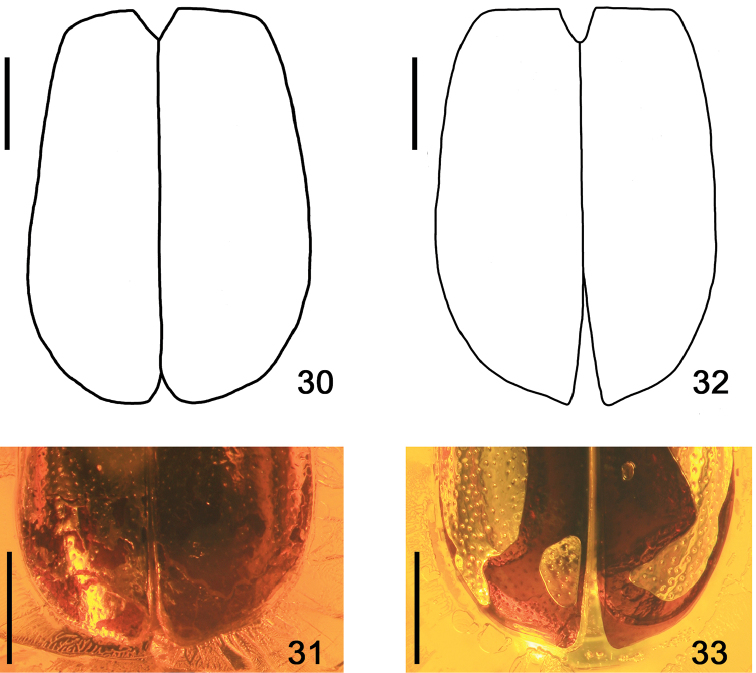
*Eusphalerumkanti* sp. nov. (holotype, male: **30, 31** paratype, female: **32, 33**): **30, 32** elytra (schematic drawings) **31, 33** apical part of elytra. Scale bars: 0.3 mm.

**Figures 34–44.**
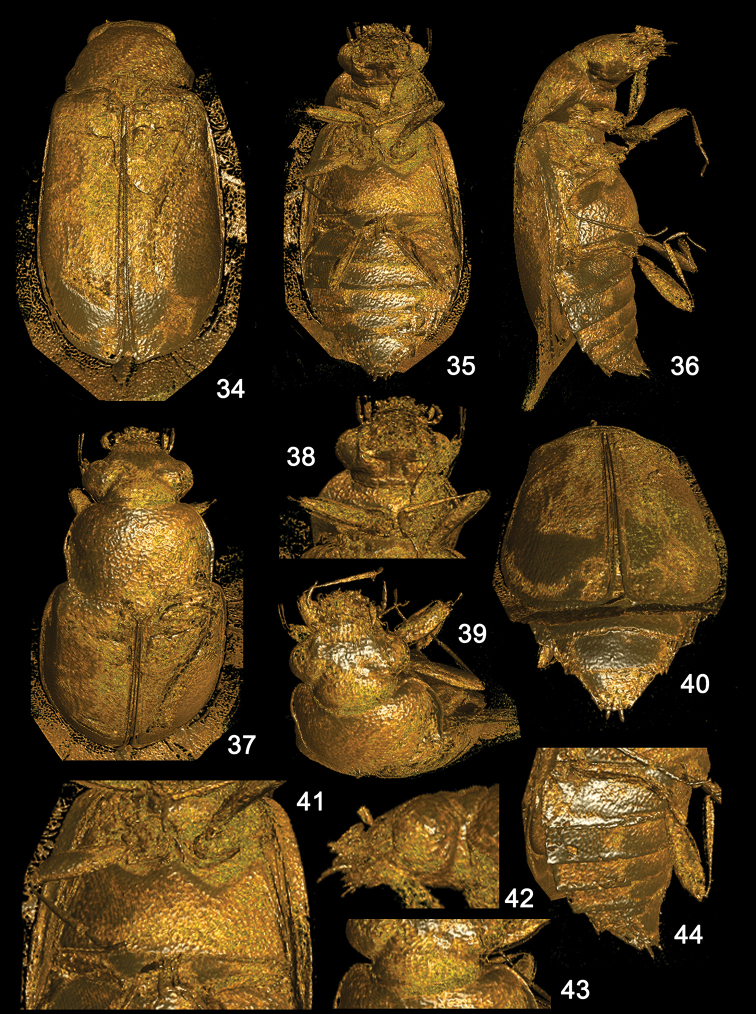
*Eusphalerumkanti* sp. nov., paratype, reconstructions from x-ray micro-computed tomography (μ-CT) **34** habitus, dorsal view **35** habitus, ventral view **36** habitus, lateral view **37** forebody, dorsal view **38** head and prothorax, ventral view **39** head and pronotum, anterodorsal view **40** elytra and abdomen, posterodorsal view **41** pterothoracic sclerites, ventral view **42** head, lateral view **43** neck and anterior portion of pronotum, dorsal view **44** abdomen, lateroventral view. Copyright 2015 Shimadzu Corporation.

Elytra sexually dimorphic (male: Figs 30, 31; female: Figs 32, 33), distinctly longer than wide (Figs 21, 23, 24, 34) and more convex behind middle; in lateral view (Fig. 36) very long, about three times as long as pronotum, distinctly widened apicad from middle, reaching middle of abdominal tergite VI, with widely rounded apical angles (Fig. 40). Punctation of elytra larger and significantly denser than that on pronotum, markedly smaller on parascutellar portion and along suture, sparser on apical portion, larger and coarser in lateroapical and medioapical portions (Figs 21, 23, 24, 31, 33).

Legs with relatively wide femora (Figs 22, 23, 25, 26, 35, 36), tibiae thin, gradually widened apicad, about as long as femora, covered by elongate setae, markedly stronger on lateral margin (Figs 22, 23, 29, 36, 44); tarsomeres 1–4 with dense distinctly elongate setae ventrally; apical metatarsomere long, as long as previous tarsomeres together (excluding tarsal claws) (Figs 23, 27–29, 36).

Abdomen distinctly narrower than elytra (Figs 27, 40); apical margin of tergite VII with indistinct brick-wall sculpture; abdominal tergites with sparse small punctures and no visible microsculpture (Fig. 27); sternites VII and VIII of both males and females without modifications (Fig. 44).

Male. Elytra as in Figure 24; apical margin of elytra widely rounded (Figs 30, 31). Apical margin of abdominal tergite VIII somewhat straight. Apical margin of abdominal sternite VIII widely rounded (Fig. 27).

Female. Elytra as in Figure 21; apical margin of elytra distinctly prolonged at sutural apex (Figs 32, 33). Apical margin of abdominal tergite VIII and sternite VIII (Figs 27, 40) straight. Genital segment with markedly elongate gonocoxites and very small styli (Figs 27, 40).

######### Etymology.

Patronymic, the species is named in honour of the great German philosopher Immanuel Kant (1724–1804), the author of the doctrine of transcendental idealism.

######### Remarks.

The paratype of *Eu.kanti* sp. nov. was visualised three-dimensionally using a micro-CT scan. Although the result was not very satisfactory, we could observe the fossil from multiple additional angles (Figs 34, 44). Based on this scan, we could describe more characters that were not visible with light microscopy. The fossil was assigned to the tribe Eusphalerini and genus *Eusphalerum* based on the general shape of the body, shapes and length of short and slightly widened tarsomeres 1–4, with dense and elongate ventral setae, together about as long as apical tarsomere, and shape of the elytra of female slightly longer than that of male, with prolonged portion at sutural apex (Figs 32, 33). This floricolous genus contains 260 valid species (Zanetti 2014) distributed in the Holarctic Region. Earlier, the genus was subdivided into two subgenera: *Eusphalerum* and *Pareusphalerum* Coiffait, 1959 (Zanetti 1987), but because several species of sensu stricto and *Pareusphalerum* were overlapping in some morphological characters, the latter was synonymized with the nominotypical taxon (Tronquet and Zanetti 2008). Based on general morphological features of the aedeagus, female accessory sclerite and, in some cases, shapes of the modified apical abdominal sternites, several species groups have been erected for many species of the genus (e.g. Zanetti 1987, 1993, 2014). However, to date, this diverse genus remains insufficiently studied globally and is in need of further phylogenetic revision because of unclear relations between both species groups and the tribe Eusphalerini with related Omaliini.

The new species is difficult to compare with extant species as they typically differ from each other by the morphology of the aedeagus and female genital structures. However, based on the shape of the strongly elongate and dimorphic elytra, *Eu.kanti* sp. nov. is like members of the following species groups: North American *convexum* (Zanetti 2014; four species distributed in Canada and USA) and western Palaearctic *amplipenne* (Zanetti 1993; one species known from Turkey), *longipenne* (Zanetti 1987; six species distributed in Middle and South Europe), *montivagum* (Zanetti 1987, 1992, 1993, 2004, 2012a; 10 species distributed in Central and Southern Europe and Turkey) and *anale* (Tronquet and Zanetti 2001; three species from the central-western part of Europe). The new species differs from the *convexum* group by the presence of the postocular carina, by the dorsal portion of head without visible impressions, by the shape of the apical tarsomere slightly longer than that in species of *convexum* group and by the abdominal sternite VII of male without modifications. It differs from the *amplipenne* group by its somewhat smaller and darker body, sparser punctation of the forebody and shape of metatarsus of male, slightly curved in *Eu.amplipenne* (see Zanetti 1993: fig. 13). The new species shares similar length of the body and postocular carina with some species of the *longipenne* group, but differs by the darker body and longer apical tarsomeres. Based on the dark body, general characters of punctation and microsculpture of head and pronotum, *Eu.kanti* sp. nov. is somewhat like some species of the *montivagum* and *anale* groups, for example Southern European *Eu.schatzmayri* (Koch, 1938), *Eu.anale* (Erichson, 1840), *Eu.brandmayri* (Zanetti, 1981), and *Eu.coiffaiti* Nicolas, 1974, but it differs by the larger body (body length of members of the *montivagum* and *anale* groups varies from 1.50 to 2.50 mm) and more transverse pronotum. From all these groups, *Eu.kanti* sp. nov. differs by the absence of distinct grooves in front of the ocelli and elongate antennomeres 2–4 (Fig. 28).

###### Tribe Omaliini MacLeay, 1825

####### Genus *Paraphloeostiba* Steel, 1960

**Type species.***Paraphloeostibamarianicola* Steel, 1960.

######## † Paraphloeostiba morosa

Taxon classificationAnimaliaColeopteraStaphylinidae

Shavrin & Yamamoto sp. nov.

http://zoobank.org/722E3364-B487-4F94-8BA6-5A38BD4A1C00

Figures 5 , 6 , 45–46 , 47, 48 , 49–53

######### Type materials examined.

Holotype (female), FMNHINS-3260632, complete specimen as inclusion in a piece of small yellow Baltic amber, 15.6 mm × 13.1 mm × 4.0 mm in size (Figs 5, 6), with the following labels: “14[printed] 11[handwritten]- SYAC 00 [printed]06 [handwritten] | Baltic / Burmite | Other: | Larva / Adult | Omaliinae [handwritten] | Kalini[n]grad [handwritten] | Shûhei Yamamoto’s | Amber Collection” <large rectangular label, printed>, “Kaliningrad, RUSSIA | Shûhei Yamamoto’s | Amber Collection | (SYAC0006)” <small rectangular label, printed>, “[FMNH barcode at left side of label] FMNHINS | 3260632 | AMBER [handwritten] | FIELD MUSEUM | Wet” <small rectangular label, printed>, “HOLOTYPE | *Paraphloeostiba* | *morosa* sp. nov. | Shavrin A. & Yamamoto S. des. 2019” <red rectangular label, printed> (FMNH).

######### Preservation.

The specimen is located at an angle with the head somewhat deeper in the amber piece (Figs 5, 6); the specimen is clearly visible from both dorsal and ventral sides. Syninclusions: round and elongate air bubbles near the specimen on from the ventral side of its body, and elongate piece of plant material located close to the dorsal surface of amber piece near the beetle.

######### Locality and horizon.

Baltic amber from Yantarny, Kaliningrad, westernmost Russia; mid-Eocene (ca 44 Ma; Wappler 2005).

######### Description.

Measurements: HW: 0.36; HL: 0.29; OL: 0.17; AL: 0.51; PML × PMW (III, IV): III: 0.03 × 0.03, IV: 0.06 × 0.02; PL: 0.31; PW: 0.74; ESL: 0.52; EW: 0.77; MTbL: 0.38; MTrL: 0.15 (I–IV: 0.07; V: 0.08); AW: 0.75; TL: ~1.80. Antennomeres with lengths × widths: 1: 0.07 × 0.03; 2: 0.05 × 0.02; 3: 0.05 × 0.01; 4: 0.03 × 0.02; 5: 0.04 × 0.02; 6–7: 0.03 × 0.03; 8: 0.03 × 0.04; 9–10: 0.04 × 0.05; 11: 0.10 × 0.05.

**Figures 45, 46.**
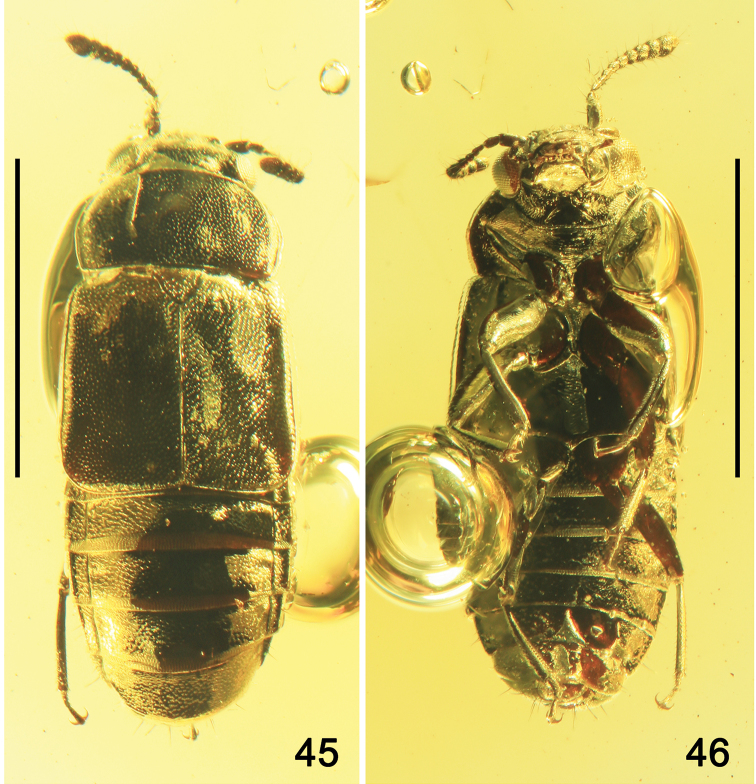
Habitus of *Paraphloeostibamorosa* sp. nov. **45** dorsal view **46** ventral view. Scale bars: 1.0 mm.

**Figures 47, 48.**
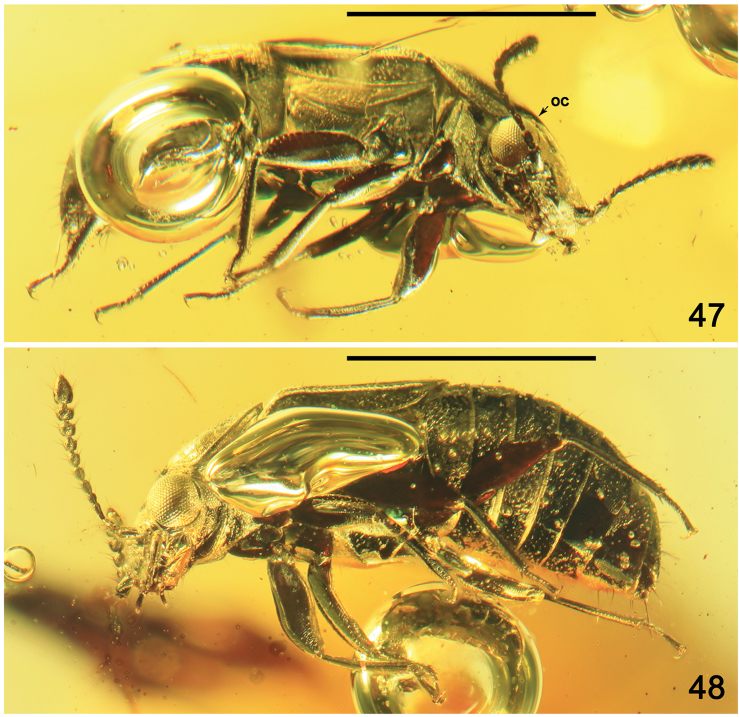
Habitus of *Paraphloeostibamorosa* sp. nov., lateral view. Abbreviation: oc = ocellus. Scale bars: 1.0 mm.

Body moderately wide, glossy (Fig. 45), black, with mouthparts, femora, and apical parts of abdominal tergites reddish-brown, and tarsi yellow-brown. Body laterally as in Figures 47 and 48. Body without visible microsculpture and setation except of paratergites and abdominal tergite VIII with long erect setae (Fig. 48).

**Figures 49–53.**
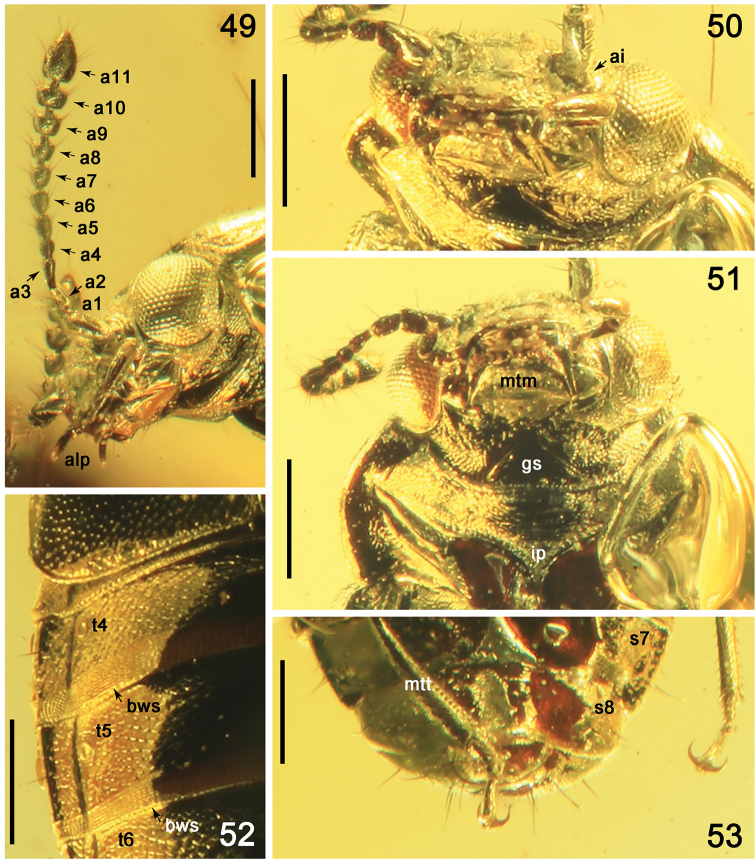
*Paraphloeostibamorosa* sp. nov. **49** head and antenna, lateral view **50** head, anteroventral view **51** head and prothorax, ventral view **52** apical part of elytron and abdominal tergites IV–V, dorsal view **53** apex of abdomen and hind legs, ventral view. Abbreviations: a1–a11 = antennomeres 1–11; ai = antennal insertion; alp = apical labial palpomere; bws = brick-wall sculpture on intersegmental membrane; gs = gular suture; ip = intercoxal process; mtm = mentum; mtt = metatibia; s7–s8 = sternites VII–VIII; t4–t6 = tergites IV–VI. Scale bars: 0.2 mm.

Head 1.2 times as wide as long, with slightly convex posterior portion, dense and small punctation and postocular carina (Fig. 49). Head laterally as in Figure 49, anteroventrally as in Figure 50, and ventrally as in Figure 51. Eyes large, with medium-sized facets, broadly convex, with distinct infraorbital carina (Figs 49–51). Ocelli moderately large, situated at level of posterior third of eyes, distance between ocelli about twice as long as distance between ocellus and medial margin of eye (Fig. 45). Labrum with widely rounded apical margin (Figs 46, 51). Apical maxillary palpomere distinctly longer and slightly narrower than penultimate, swollen in middle and elongate, from middle gradually narrowing toward rounded apex (Figs 49–51). Submentum large, trapezoidal; apical labial palpomere elongate, from middle narrowing apicad (Fig. 49). Gular sutures with markedly rounded posterior parts widely separated from each other below level of posterior margins of eyes (Figs 50, 51). Gena with rugose isodiametric microsculpture (Figs 50, 51). Antenna moderately short, exceeding basal portion of pronotum, with sparse elongate setation; basal antennomere swollen, more than twice as long as wide, antennomere 2 elongate, slightly widened apicad, 3 thin, as long as 2, 4, and 5 markedly widened apicad, 6 and 7 as long as wide, 8–10 slightly transverse, apical antennomere wide, from apical third slightly narrowing toward rounded apex (Figs 45–49).

Pronotum with slightly convex surface, markedly transverse, more than twice longer than broad, twice wider than head, from middle slightly more narrowed anterad than posterad, with widely rounded anterior and scarcely rounded posterior angles; apical margin widely rounded, distinctly shorter than somewhat concave posterior margin; paramedian longitudinal impressions indistinct, wide and long, occupying most of middle portion; lateral margins narrowly emarginate, with indistinctly concave laterobasal margins; posterior angles without depressions (Fig. 45). Dorsal surface of pronotum with more or less regular small and dense punctation, distinctly denser than in posterior portion of head (Fig. 45). Prosternum with widely open procoxal fissures, exposing trochantins, and very long intercoxal process, with acute apex reaching apical part of procoxae (Figs 46, 51). Median part of mesoventrite somewhat convex, with very long acute intercoxal process, reaching more than halfway along the length of the mesocoxae and moderately wide apex of metaventral process (Fig. 46). Scutellum large and triangular, with rounded apex and dense punctation in apical part (Fig. 45). Metaventrite with moderately wide metacoxal cavities (Fig. 46). Median part of prosternum and metaventrite with indistinct and sparse small punctures; mesanepisternum with diagonal microsculpture; median portions of prosternum and metaventrite, including intercoxal processes, with transverse meshes (Fig. 46).

Elytra evidently flattened, 1.4 times as wide as long, 1.6 times as long as pronotum, with moderately parallel lateral sides (Fig. 45), with widely rounded apical angles (Fig. 52), reaching apical margin of abdominal tergite III, with apical margins slightly oblique toward suture (Fig. 45). Punctation as that in pronotum, slightly sparser in basal portion and near scutellum.

Legs moderately long and slender, with wide femora and slender tibiae, gradually widened apicad, covered by elongate setae on both inner and outer margins and with a few strong setae on outer margins (Figs 46–48); tarsi short, with small setae on tarsomeres 1–4, apical metatarsomere slightly longer than preceding tarsomeres together; tarsal claws simple, widely curved and elongate (Figs 46, 53).

Abdomen convex, slightly narrower than elytra, with wide brick-wall sculpture on intersegmental membranes between tergites III–VI (Fig. 52) and sternites III–VI (Fig. 45). Abdominal tergites with moderately dense and deep small punctation and distinct net-like microsculpture (Fig. 52); abdominal sternites with indistinct sparse punctation, with shallow isodiametric microsculpture (Figs 45, 52).

**Figures 54–56.**
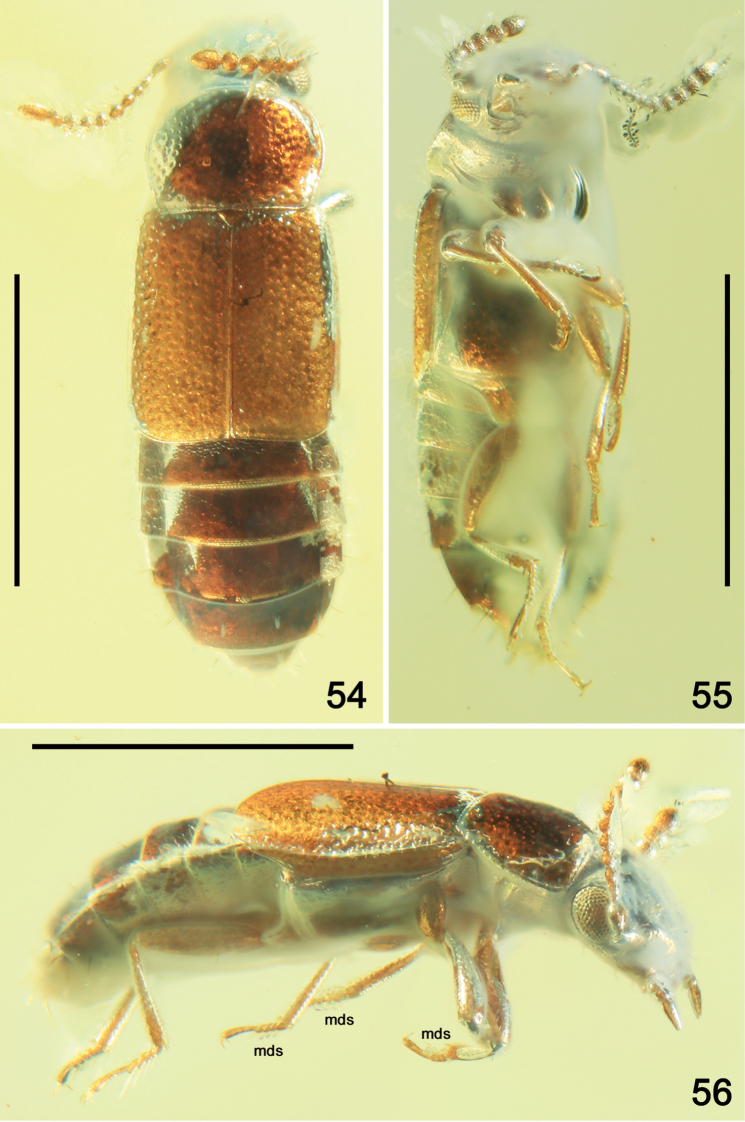
Habitus of *Phyllodrepadaedali* sp. nov. **54** dorsal view **55** lateroventral view **56** lateral view. Abbreviation: mds = modified setae. Scale bars: 1.0 mm.

Male unknown.

Female. Apical margin of abdominal tergite VIII rounded. Apical margin of abdominal sternite VIII broadly concave (Fig. 53). Genital segment with moderately wide apical portions of gonocoxites; shape and length of styli invisible, each with very long seta (Figs 47, 48).

######### Etymology.

The specific epithet is the Latin adjective *morosus*, -*a*, -*um* (strange). It refers to somewhat broad body with markedly transverse pronotum of the new species.

######### Remarks.

Based on the shape of body and maxillary palpomeres (see also Zanetti 2012: fig. 55l), slightly convex pronotum, punctation and microsculpture of the surface of body, the fossil presumably belongs to the genus *Paraphloeostiba*. The genus was erected by Steel (1960a) and was compared with *Phloeostiba* Thomson, 1858 and *Phloeonomus* Heer, 1839. It differs from *Phloeostiba* by the shape of short maxillary palpomere 3 and relatively elongate apical palpomere, and from *Phloeonomus* by a different shape of ligula and maxillary palp (for details see Steel 1960a). *Paraphloeostiba* includes more than 30 species distributed in the Palaearctic, Madagascan, Nearctic, and predominantly in Oriental, Australian, and Oceanic regions (Steel 1960a; Herman 2001; Shavrin and Smetana 2016; Shavrin 2017b); one species, *P.gayandahense* (W.J. MacLeay, 1873) is widely adventive around the world to New Zealand, several countries of Europe, and the USA (Herman 2001). The new species is difficult to compare reliably with known species as these mostly differ by the structure of the aedeagus, and shapes of accessory sclerite and spermatheca. The apical antennomeres of *P.morosa* sp. nov. are slightly transverse, beginning with antennomere 8 (Figs 48, 49) while other known species have transverse antennomeres beginning with 6 or 7. Based on the punctation and microsculpture of the pronotum and shape of antennomere 10 (Fig. 49), the new species is similar to *P.specularis* (Bernhauer, 1915), known from New Britain (Bismarck Archipelago of Papua New Guinea) but differs by the somewhat larger and wider body, the absence of laterobasal pronotal depressions, and the more transverse pronotum (Fig. 45). Based on the shape and coloration of the body, similar punctation, pronotum without depressions on basal portions, and somewhat convex mesoventrite, *P.morosa* sp. nov. is also similar to *P.electrica*Zanetti et al., 2016, recently described from Baltic amber, from which it differs by the wider body with more transverse pronotum, as well as elongate antennomeres 4–7, and wide apical and penultimate palpomeres.

*Paraphloeostiba* requires revision due to unclear morphological boundaries between described species and related genera, as well as many undescribed species from the Oriental and Australian regions deposited in institutional and private collections. The new species is tentatively attributed to this genus, making it the second extinct representative of the genus after *P.electrica*.

####### Genus *Phyllodrepa* Thomson, 1859

**Type species.***Staphylinusfloralis* Paykull, 1789

######## † Phyllodrepa daedali

Taxon classificationAnimaliaColeopteraStaphylinidae

Shavrin & Yamamoto sp. nov.

http://zoobank.org/BDE372B6-F773-433D-B98E-C0E272FB8FA7

Figures 7–9 , 54–56 , 57–64

######### Type materials examined.

Holotype (male), FMNHINS-3260629, complete specimen as inclusion in very small piece of light yellow Baltic amber, 9.3 mm × 5.9 mm × 2.9 mm in size (Figs 7–9), with the following labels: “16 [printed] 02 [handwritten] SYAC 0 [printed] 294 [handwritten] | Baltic / Burmite | Other: | Larva / Adult | Omalium sp. [handwritten] | Omaliinae [handwritten] | Yantarny, Kaliningrad [handwritten] | Shûhei Yamamoto’s | Amber Collection” <large rectangular label, printed>, “[FMNH barcode at left side of label] FMNHINS | 3260629 | AMBER [handwritten] | FIELD MUSEUM | Wet” <small rectangular label, printed>, “HOLOTYPE | *Phyllodrepa* | *daedali* sp. nov. | Shavrin A. & Yamamoto S. des. 2019” <red rectangular label, printed> (FMNH).

######### Preservation.

The specimen is relatively well preserved and many details are visible, from the dorsal, ventral and lateral sides (Figs 7–9). However, most body parts, except the dorsal surface of the head, are covered with cloud of milky substance, especially most of the ventral side.

######### Locality and horizon.

Baltic amber from Yantarny, Kaliningrad, westernmost Russia; mid-Eocene (ca 44 Ma; Wappler 2005).

######### Description.

Measurements: HW: 0.32; HL: 0.22; OL: 0.11; AL: 0.52; PML × PMW (III, IV): III: 0.02 × 0.02, IV: 0.07 × 0.02; PL: 0.35; PW: 0.48; ESL: 0.56; EW: 0.51; MTbL: 0.31; MTrL: 0.20 (I–IV: 0.08; V: 0.12); AW: 0.50; TL: ~1.80. Antennomeres with lengths × widths: 1: 0.08 × 0.03; 2: 0.06 × 0.02; 3–4: 0.05 × 0.02; 5: 0.04 × 0.02; 6: 0.04 × 0.03; 7: 0.03 × 0.03; 8–10: 0.03 × 0.04; 11: 0.08 × 0.04.

**Figures 57–64.**
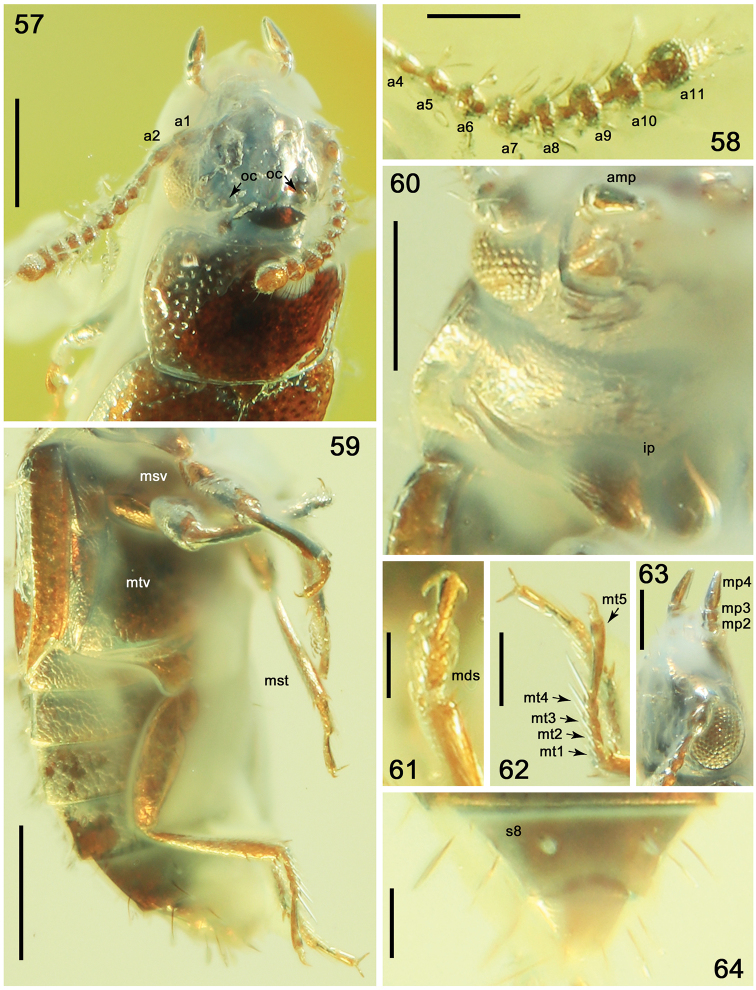
*Phyllodrepadaedali* sp. nov. **57** head and pronotum, laterodorsal view **58** left antenna, ventral view **59** thorax, legs and abdomen, lateroventral view **60** head and prothorax, ventral view **61** protarsus, dorsal view **62** hind tarsi, lateral view **63** head and maxillar palpi, dorsolateral view **64** apex of abdomen, ventral view. Abbreviations: a1–a11 = antennomeres 1–11; amp = apical maxillary palpomere; ip = intercoxal process; mp2–mp4 = maxillary palpomeres 2–4; mds = modified setae; mst = mesotibia; msv = mesoventrite; mt1–mt5 = metatarsomeres 1–5; mtv = metaventrite; oc = ocellus; s8 = sternite VIII. Scale bars: 0.1 mm (**58, 61–63**), 0.2 mm (**57, 60, 64**), 0.3 mm (**59**).

Body elongate and slightly convex, glossy (Fig. 54), reddish-brown, with darker head and abdomen; mouthparts, antennae, legs and apical margins of abdominal sclerites yellow-brown. Body lateroventrally as in Figure 55 and laterally as in Figure 56. Lateral margins of pronotum (Figs 54, 57), paratergites and abdominal tergite VIII (Fig. 64) with several long erect setae.

Head 1.4 times as wide as long, with slightly convex median portion and slight oval lateroapical impressions (Fig. 57), with sparse, small and moderately deep punctation, with shallow postocular carina. Eyes large and broadly convex (Figs 55–57, 60). Ocelli large and convex, situated at level of posterior margins of eyes, distance between ocelli much more than twice as long as distance between ocellus and posterior margin of eye; grooves in front of ocelli present, moderately deep and short (Fig. 57). Apical segment of maxillary palp significantly longer than small penultimate segment, from swollen middle gradually narrowing apicad (Figs 56, 57, 63). Antenna moderately short, just surpassing basal margin of pronotum, with sparse very long setae on antennomeres 5–11; basal and antennomere 2 swollen and elongate, 3 and 4 narrow and elongate, 5 ovoid, 6 and 7 slightly transverse and 8–10 distinctly transverse, apical antennomere large, strongly narrowing from about middle apicad (Figs 54–58).

Pronotum slightly convex, without longitudinal impressions, 1.3 times as wide as long, 1.5 times as wide as head, from middle distinctly more narrowed posterad than apicad, with widely rounded anterior and obtuse posterior angles; apical margin widely rounded, slightly shorter than somewhat straight posterior margin; lateral margins slightly sinuate posteriorly, narrowly emarginate and finely crenulate; lateroposterior portions with indistinct, moderately wide impressions (Figs 54, 57). Dorsal surface of pronotum without visible microsculpture between punctures, with dense, very large and deep punctation, markedly sparser in lateral and smaller in apical and basal portions (Figs 54, 57). Prosternum with wide procoxal fissures and moderately short prosternal process, with acute apex (Fig. 60). Scutellum moderately large, triangular, with somewhat rounded apex, without visible punctures or microsculpture (Fig. 54).

Elytra slightly convex, longer than wide, 1.6 times as long as pronotum, reaching basal margin of abdominal tergite IV, with somewhat parallel lateral sides and widely rounded lateroapical angles, with sutural apices truncate to very oblique (Fig. 54). Punctation as that in pronotum, but shallower and somewhat smoothed on apical portion, smaller and sparser on basal and apical portions. Surface between punctures with shallow dense isodiametric microsculpture.

Legs long and slender, similar in shape, with moderately wide femora; tibiae slender, gradually widened apicad, covered by elongate setae, denser and stronger on inner margins, and with a few strong spines near apex and additional spine on outer margin in apical third (Figs 55, 56, 59); tarsi long, with apical metatarsomere distinctly longer than previous tarsomeres together (Figs 59, 61, 62); protarsus as in Figure 61, with long tenent setae (probably only in males); tarsal claw simple (Figs 61, 62).

Abdomen markedly convex, slightly narrower at base than elytra; wing-folding patches in middle of tergite IV and/or V not visible; intersegmental membranes between tergites IV–VII with brick-wall sculpture, apical margin of tergite VII with indistinct very narrow palisade fringe (Fig. 54). Abdominal tergites without visible punctation, with large distinct transverse microsculpture.

Male. First four protarsomeres wide (Figs 55, 59, 61); ventral surface of protarsomeres 1–4 with several rows of modified tenent setae (not all details visible) consisting of internal rows formed by markedly elongate setae with leaf-shaped apical parts (Figs 55, 56, 59, 61); ventral surface of mesotarsomeres 1–4 with two rows of elongate setae with broadened apical parts as that in protarsomeres 1–4 but without additional internal rows (Figs 55, 56); metatarsi as in Figure 62. Apical margin of abdominal tergite VIII straight.

Female unknown.

######### Etymology.

The specific epithet is the Latinized name of *Daedalus*, -*i*, *m*, the Greek architect of the times of Theseus and Minos, and father of Icarus.

######### Remarks.

In external characters such as proportions of the body, antennomeres, and maxillary palpomeres, and, more substantially, by the proportions of tarsi with elongate apical tarsomere, the fossil undoubtedly belongs to the tribe Omaliini. Based on the triangular and elongate apical maxillary palpomere, shape of slightly convex head and slightly transverse antennomere 7, presence of two small depressions between bases of antennae, short grooves (dorsal tentorial pits) in front of the ocelli, and shape of the moderately convex pronotum with slightly sinuate lateral margins in front of obtuse posterior angles, the new species belong to the *Phyllodrepa* complex, specifically to the genus *Phyllodrepa*. *Phyllodrepa* includes about 30 species distributed in Palaearctic, Nearctic, and Neotropical regions (Newton et al. 2000; Herman 2001; Schülke and Smetana 2015). The genus requires a worldwide revision and apparently includes some taxa that belong to other related genera (Shavrin 2016; Zanetti et. al. 2016). *Phyllodrepadaedali* sp. nov. and *Ph.icari* sp. nov., described below, are species with a very small and pale body that reminds of some Palaearctic species of the genus *Dropephylla* Mulsant & Rey, 1880, that for a long time had been regarded as a subgenus of *Phyllodrepa*. Nevertheless, species of *Dropephylla* differ by the absence of grooves in front of ocelli and microsculpture on the elytra, wider apical maxillary palpomere, oval antennomere 4, by the presence of moderately defined short and rounded temples, shorter apical tarsomere, and other morphological characters that were considered in the revision of the Palaearctic fauna of the genus by Jászay and Hlaváč (2006). Although faintly crenulate lateral margins of the pronotum (Fig. 57) are similar to those in *Dialycera* Ganglbauer and *Hapalaraea* Thomson (Zanetti 1987, 2012b; Zanetti et al. 2016), they are also known to the first author in European *Phyllodrepapuberula* Bernhauer, 1903 and some little-known species distributed in the eastern Palaearctic Region. Despite this, the new species can not be reliably associated with any extant species of the genus due to its unique morphological characters and the fact that most species differ only by the external structure of the aedeagus. Both new species of *Phyllodrepa* described herein differ from the more ancient Transbaikal †*Eophyllodrepa* Ryvkin from the Middle-Upper Jurassic of Novospasskoe (Ryvkin 1985) and †*Daidromus* Ryvkin from the Upper Jurassic of Daya (Ryvkin 1990), by the same morphological characters as in *Ph.electrica* (see Zanetti et al. 2016). Based on the small body, shape of head, general shape of apical antennomeres, and pronotum with finely crenulate lateral margins, *Ph.daedalum* sp. nov. is similar to *Ph.antiqua* Zanetti, Perreau & Solodovnikov, 2016, which was recently described from Baltic amber (Zanetti et. al 2016). It is also similar to *Ph.icari* sp. nov. (see below), based on the crenulate lateral pronotal margins, pale body, and large and deep punctation of the elytra. It differs from *Ph.antiqua* by the smaller, paler and slightly more convex body (Figs 54, 56), coarser and deeper punctation of pronotum (Figs 54, 57) and elytra (Fig. 54), and elongate antennomeres 2–5 (Fig. 58), and from *Ph.icari* sp. nov. by the darker abdomen, wider apical maxillary palpomere (Fig. 57), shape of anterior angles of the pronotum not protruded apicad (Fig. 57), denser punctation of the pronotum, less transverse head and pronotum (Fig. 57), and longer antennomeres 4–5 and 11 (Fig. 58). From both these species it differs by longer elytra, and from *Ph.antiqua* by the presence of modified setae on tarsomeres 1–4 of front and middle legs of the male.

A remarkable morphological feature of *Ph.daedali* sp. nov. is the presence of modified rows of elongate setae (Figs 55, 56, 61) with leaf-shaped apical parts on ventral surface of pro- and mesotarsomeres 1–4, described earlier as disco-setae (Stork 1980) or clavate adhesive setae (Smetana 1987). Similar structures were observed in species of the Oriental genera *Xanthonomus* Bernhauer by Steel (1955: fig. 6), *Prosopaspis* Smetana (Smetana 1987: fig. 22), *Duocalcar* Peris & Thayer, 2014 (at least protarsi), and *Paraphloeostiba* (Steel 1960a).

######## † Phyllodrepa icari

Taxon classificationAnimaliaColeopteraStaphylinidae

Shavrin & Yamamoto sp. nov.

http://zoobank.org/C1EC61D0-CD67-48CC-92C0-07489AE05B8A

Figures 10 , 11 , 65–66 , 67–72

######### Type materials examined.

Holotype (female), FMNHINS-3260628, complete specimen as inclusion in small rectangular light yellow Baltic Amber, 19.2 mm × 7.9 mm × 5.1 mm in size (Figs 10, 11), with the following labels: “15[printed]04[handwritten] – SYAC 00[printed]94[handwritten] | Baltic / Burmite | Oher: | Larva / Adult | Omaliinae[handwritten] | Baltic Sea Coast, [handwritten] | close to the Wisla Riv. | Estuary, Poland[handwritten] | Shûhei Yamamoto’s | Amber Collection” <large rectangular label, printed>, Nadewca / Sender: | Artur Michalski” <large light yellow rectangular label, handwritten>, “[FMNH barcode at left side of label] FMNHINS | 3260628 | AMBER [handwritten] | FIELD MUSEUM | Wet” <small rectangular label, printed>, “HOLOTYPE | *Phyllodrepa* | *icari* sp. nov. | Shavrin A. & Yamamoto S. des. 2019” <red rectangular label, printed> (FMNH).

######### Preservation.

The specimen is in relatively good condition and best visible from the dorsal side of the body, except the head and right antennomeres 9–11, and with head visible from the narrow side of the amber piece (Fig. 10). The details of the ventral side of inclusion are not visible except for the apical antennomeres, middle and hind legs, and a part of the mesothoracic segment and abdominal sternites (Figs 66, 69, 72). Syninclusion: imago of small Diptera about 1.20 mm in length.

**Figures 65, 66.**
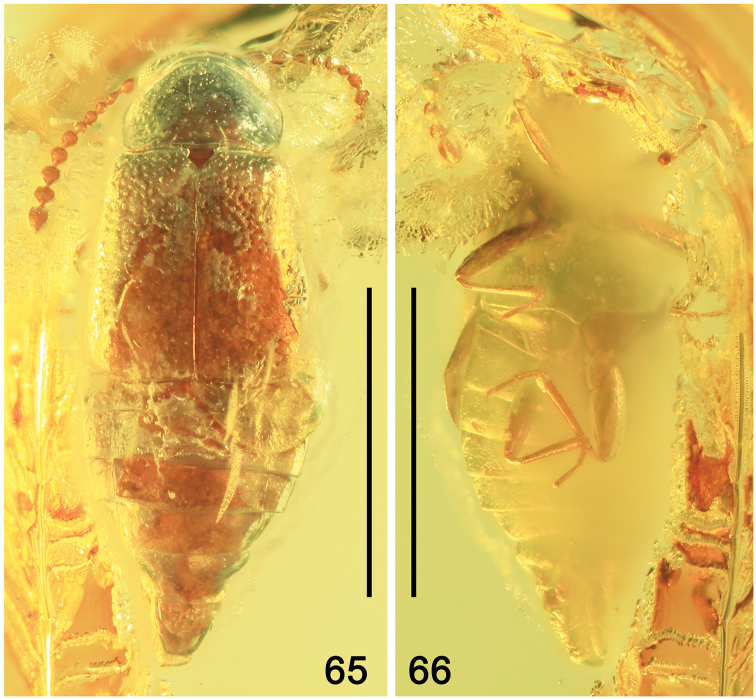
Habitus of *Phyllodrepaicari* sp. nov. **65** dorsal view **66** ventral view. Scale bars: 1.0 mm

######### Locality and horizon.

Baltic amber from Baltic Sea Coast, close to the estuary of Wisła River, Gdańsk, Poland; mid-Eocene (ca 44 Ma; Wappler 2005).

######### Description.

Measurements: HW: 0.31; HL: 0.18; OL: 0.11; AL: 0.50; PML × PMW (III, IV): III: 0.03 × 0.02, IV: 0.05 × 0.01; PL: 0.25; PW: 0.46; ESL: 0.58; EW: 0.66; MTbL: 0.16; MTrL: 0.11 (I–IV: 0.05; V: 0.06); AW: 0.66; TL: ~1.80. Antennomeres with lengths × widths: 1: 0.08 × 0.03; 2: 0.05 × 0.02; 3: 0.04 × 0.02; 4–5: 0.03 × 0.02; 6: 0.02 × 0.02; 7: 0.04 × 0.03; 8: 0.04 × 0.04; 9: 0.05 × 0.05; 10: 0.05 × 0.06; 11: 0.07 × 0.06.

In general appearance, body (Fig. 65) and legs as in *Ph.daedali* sp. nov., reddish-brown, with darker head and pronotum; mouthparts, antennae and legs yellow-brown. Body ventrally as in Figure 66.

**Figures 67–72.**
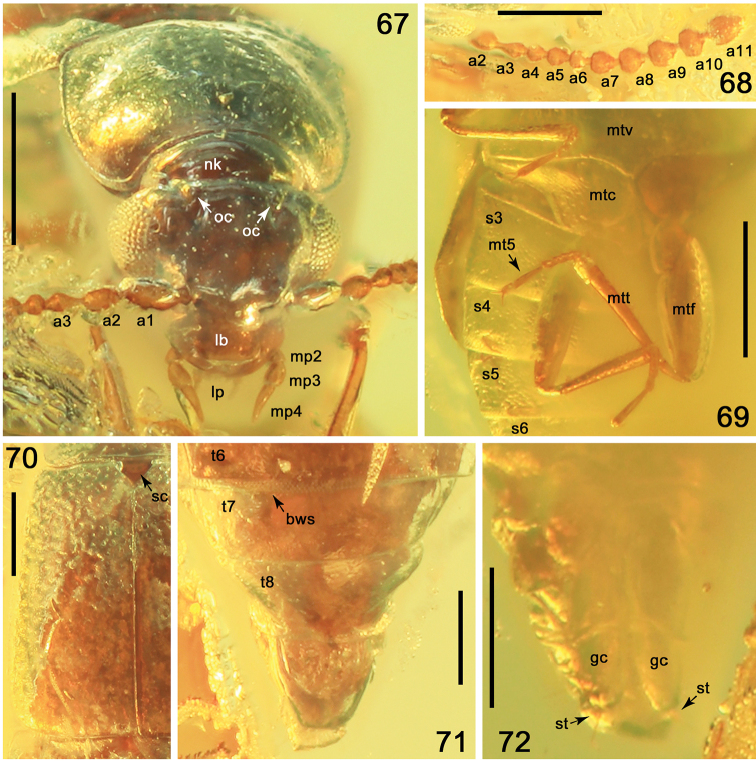
*Phyllodrepaicari* sp. nov. **67** head and pronotum, anterodorsal view **68** left antenna, dorsal view **69** hind legs and abdomen, ventral view **70** left elytron, dorsal view **71** apex of abdomen, dorsal view **72** apex of abdomen, ventral view. Abbreviations: a1–a11 = antennomeres 1–11; bws = brick-wall sculpture on intersegmental membranes; gc = gonocoxite; lb = labrum; lp = labial palpi; mp2–mp4 = maxillary palpomeres 2–4; mt5 = metatarsomere 5; mtf = metafemur; mtt = metatibia; mtc = metacoxa; mtv = metaventrite; nk = neck; oc = ocellus; s3–s6 = sternites III–VI; sc = scutellum; st = stylus; t6–t8 = tergites VI–VIII. Scale bars: 0.2 mm.

Head transverse, 1.7 times as wide as long, with slightly convex median portion (Fig. 67); punctation and postocular carina invisible. Eyes very large and broadly convex (Fig. 67). Ocelli large and convex, situated almost at level of posterior margins of eyes, distance between ocelli about twice as long distance between ocellus and medial margin of eye; grooves in front of ocelli very short and moderately deep (Fig. 67). Labrum wide and transverse, with slightly rounded apical margin (Fig. 67). Apical segment of maxillary palp narrow, narrowing from base toward moderately acute apex, distinctly longer and narrower than swollen penultimate segment (Fig. 67). Antenna moderately long, reaching basal third of elytra; antennomeres 1 and 2 swollen and elongate, 3 with very narrow elongate base, 4–6 ovoid, 7–9 slightly and 10 distinctly transverse, apical antennomere slightly longer than wide, strongly narrowed from about apical third (Figs 65, 68).

Pronotum transverse, 1.8 times as wide as long, 1.4 times as wide as head, from middle more narrowed posterad than anterad, with widely rounded slightly protruding anterior and obtuse posterior angles (Fig. 67); apical margin moderately widely concave, distinctly shorter than posterior margin; lateral margins narrowly marginate and slightly crenulate, more distinct posteriorly; laterobasal portions with indistinct wide impressions (Figs 65, 67). Dorsal surface of pronotum with moderately sparse, large and deep punctation, distinctly sparser in basal and apical portions, with narrow impunctate longitudinal area (Figs 65, 67). Scutellum large, with triangular apex, without punctures or microsculpture (Fig. 65).

Elytra 1.2 times as long as wide, reaching apical margin of abdominal tergite III, slightly widened apicad, with widely rounded apicolateral angles and apical margins truncate at suture (Figs 65, 70). Punctation denser, markedly larger and deeper than that on pronotum, smaller in basal and apical, and sparser in lateral portions. Surface between punctures with dense isodiametric microsculpture.

Tarsi long, with apical tarsomere markedly longer than previous tarsomeres together (Figs 66, 69).

Abdomen slightly convex, as wide as elytra or slightly wider, intersegmental membranes between tergites IV–VII with brick-wall sculpture (Fig. 65). Abdominal tergites with indistinct small and very sparse punctation and microsculpture, and with sparse and short pubescence, wing-folding patches not visible.

Male unknown.

Female. First four mesotarsomeres 1–4 without modified setae (Figs 66, 69). Apical margin of abdominal tergite VIII slightly rounded (Fig. 71). Apical margin of abdominal sternite VIII widely concave (Fig. 72). Genital segment with markedly elongate and wide gonocoxites, with very small narrow styli, each with very long seta (Fig. 72).

######### Etymology.

The specific epithet is the Latinized name of Icarus (Ikaros), son of Deaedalus in Greek mythology.

######### Remarks.

Despite the shape of antennomere 3 and the posterior angles of the pronotum, which are usual in members of the genus *Acrolocha* Thomson, in other external characters (see details above), the new species belongs to the genus *Phyllodrepa*. The fossil shares with that genus slightly protruded anterior angles of the pronotum with impressed laterobasal portions (Fig. 67), similar to those of extant North European *Ph.sahlbergi* Luze, in addition to similar coloration of the body and proportions of antennomeres 4 and 5 (Fig. 68). However, the fossil differs from that species by the significantly smaller and slightly more convex body (Fig. 65), with more prominent eyes (Fig. 67), coarser punctation of the elytra and pronotum, pronotum with less sinuate lateral margins (Figs 65, 67), less transverse antennomeres 6–10 (Fig. 68), and longer elytra (Fig. 70). Based on the shape of its small and pale body, and large and deep punctation of the elytra (Figs 65, 70), *Ph.icari* sp. nov. is similar to *Ph.daedali* sp. nov., from which it differs by the paler abdomen, narrow apical maxillary palpomere (Fig. 67), the shape of anterior angles of the pronotum protruding apicad, sparser punctation of the pronotum, distinctly transverse head and pronotum (Fig. 67), shorter antennomeres 4, 5, and 11 (Fig. 58), and shorter elytra.

####### Unnamed species

######## Eusphalerum

Taxon classificationAnimaliaColeopteraStaphylinidae

sp. 1

Figures 14–16 , 73–75 , 76–80

######### Materials examined.

2 males (no. 6, no. 9), 2 females (no. 3, no. 7), 5 unsexed specimens (no. 1, no. 2, no. 4, no. 5, no. 8), FMNHINS-3260631, complete specimens as inclusions in yellow Baltic amber 31.3 mm × 20.6 mm × 12.6 mm in size (Figs 14–16), with the following labels: “15[printed]03[handwritten]-SYAC 00[printed]8[handwritten] | Baltic / Burmite | Other: | Larva / Adult | Omaliinae 11 exs, [handwritten] | Baltic Sea Coast [handwritten] | Axel (Germany) [handwritten] | Shûhei Yamamoto’s | Amber Collection” <large rectangular label, printed>, “Axel Niggeloh” <rectangular label, printed>, “[FMNH barcode at left side of label] FMNHINS | 3260631 | AMBER [handwritten] | FIELD MUSEUM | Wet” <small rectangular label, printed>, “*Eusphalerum* | sp. 1 | Shavrin A.V. det. 2018” <rectangular label, printed> (FMNH).

######### Preservation.

The specimens are visible from one surface of the piece of amber (specimens were numbered as in Figs 14–16). One of the best preserved specimens (male, no. 6) is located dorsolaterally: the surface of the body, left antenna, and part of the abdomen in lateral view are clearly visible. Eight other specimens are present with differing degrees of visible details. A male (no. 9) is located deep in the piece of amber at the level of its median convexity; its pronotum, elytra, and abdomen are clearly visible dorsolaterally, and the antennae, mouthparts, legs and parts of the thoracic segments and abdomen are visible ventrally. A female (no. 3) is laterally oriented close to the outer surface of the amber piece; its basal antennomeres of the right antenna are partly visible, and the mouthparts, including maxillary and labial palpi, lateral side of the elytra, and partly thoracic sclerites, and legs relatively are visible. Another female (no. 7) is located laterally deep in the piece of amber and, therefore, the dorsal side of its body is visible but strongly cloudy and distorted; details of the structure of maxillary palpus, right antenna, five legs partly, some details of the thorax and abdomen, including apical portion with the genital segment are more or less visible from lateral view. An unsexed specimen (no. 1) is located dorsally near the outer surface of the piece of amber; only the pronotum and elytra are partly visible. Another unsexed specimen (no. 2) is located dorsally near the outer surface of the piece of amber near specimen no. 1; the apical segments of its right antenna, pronotum, and elytra are clearly visible. An unsexed specimen (no. 4) is located a little deeper in the piece of amber, close to specimen no. 3; its hind wings are extended and cover the entire abdomen. The body is not clearly visible except for the pronotum and elytra. An unsexed specimen (no. 5) is located dorsally near specimen no. 6, with the left antenna, posterior portion of head, pronotum, and elytra partially visible. Another unsexed specimen (no. 8) is located deep in the piece of amber, near specimen no. 9, and has its hind wings extended so as to cover the dorsal side of the abdomen; there is a milky covering on the lateral side of the body, and, therefore, the middle and hind legs are only partly visible in lateral view and the pronotum and elytra are partly visible in dorsal view. Syninclusion near outer surface of the piece of amber close to its margin: imago of Diptera about 2.80 mm length, including wings (Fig. 14).

######### Description.

Measurements (n = 9): HW: 0.67 (no. 5); HL: ?; OL: 0.18 (no. 6); AL (no. 6): 0.74; PML × PMW: ?; PL: 0.41–0.46; PW: 0.87 (no. 5); ESL: 0.83–0.96; EW: 0.71–0.77; MTbL (no. 8): 0.40; MTrL (no. 8): 0.28; AW: 0.68–0.74; TL: ~2.50–3.20. Antennomeres with lengths × widths (no. 6): 1: 0.15 × 0.05; 2: 0.08 × 0.04; 3: 0.06 × 0.03; 4–6: 0.05 × 0.03; 7: 0.05 × 0.04; 8: 0.06 × 0.04; 9 0.05 × 0.05; 10: 0.06 × 0.05; 11: 0.08 × 0.05.

**Figures 73–75.**
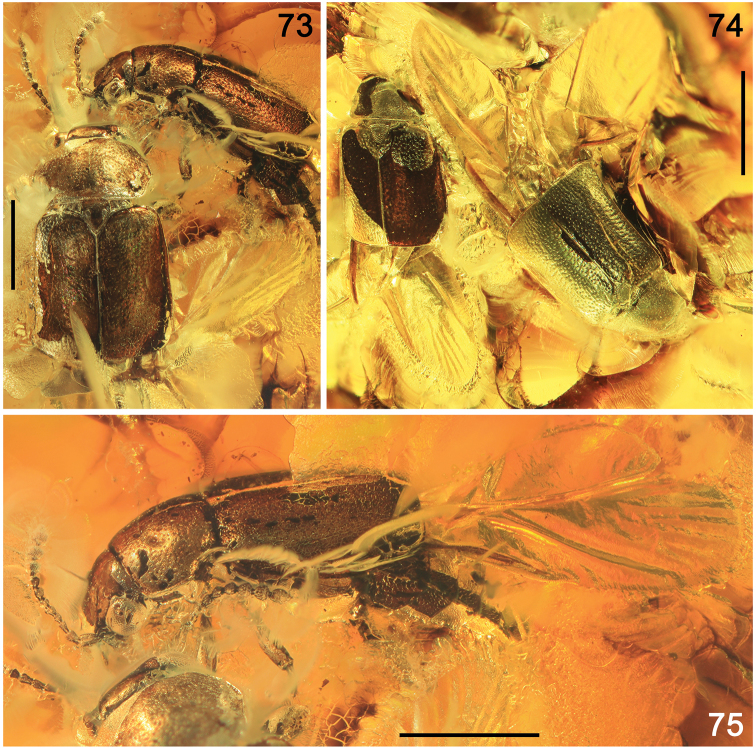
Habitus of *Eusphalerum* sp.1: **73** forebody, dorsal (specimen no. 5) and lateral (specimen no. 6) view **74** pronotum and elytra of specimens no. 1 and no. 2, dorsal view **75** body, lateral view (specimen no. 6). Scale bars: 1.0 mm.

**Figures 76–80.**
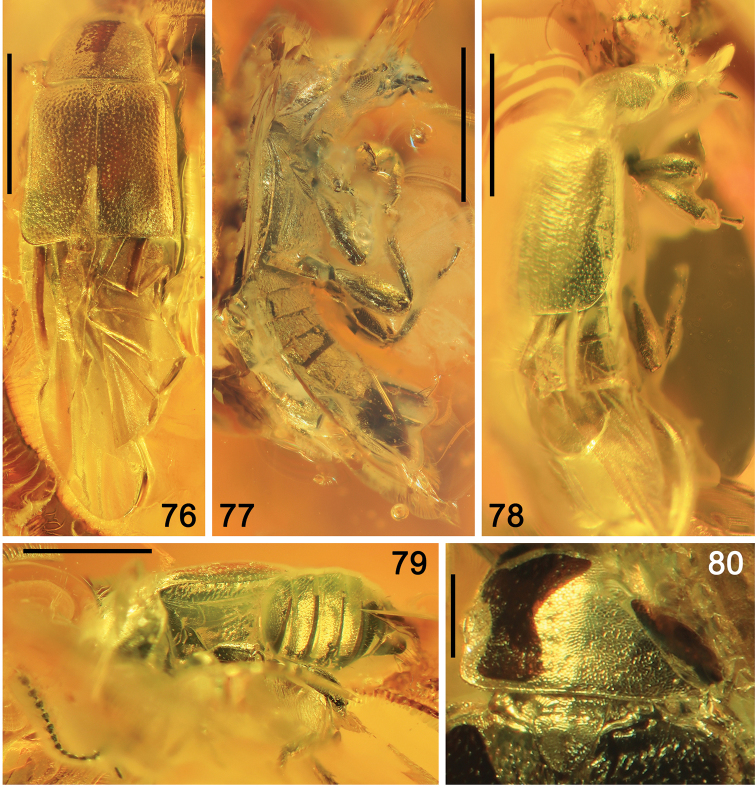
*Eusphalerum* sp. 1 **76** habitus, dorsal view (specimen no. 4) **77** habitus, lateral view (specimen no. 3) **78** habitus, dorsolateral view (specimen no. 8) **79** habitus, lateral view (specimen no. 9) **80** pronotum and scutellum, dorsal view (specimen no. 1). Scale bars: 1.0 mm (**76–79**), 0.2 mm (**80**).

Body moderately wide, convex (Figs 73–76). Body laterally as in Figures 77 and 79, dorsally as in Figs 73, 74, and 76 and dorsolaterally as in Figure 78. The specimens appear brown to black. Body glabrous, without visible setation.

Head transverse, with slightly convex middle portion, without grooves in front of ocelli (Fig. 75); punctation of posterior portion of head irregular, small, and sparse; surface between punctures with relatively large transverse fine microsculpture, distinctly larger and coarser on neck. Eyes large, widely convex (Figs 75, 77). Ocelli relatively small, slightly convex (Figs 73, 75). Apical segment of maxillary palp elongate, slightly narrower and distinctly longer than penultimate segment, from middle gradually narrowed apicad, with moderately acute apex (Fig. 77). Antenna reaching basal margin of elytra; basal antennomere markedly wide, antennomere 2 swollen and slightly elongate, 3 with thin basal portion, slightly widened apicad, antennomeres 4–6 slightly shorter than 3, 7 slightly wider than 6 and 8, 10 slightly transverse in apical portion, apical antennomere wider than penultimate segment, from apical third gradually narrowed apicad (Figs 73, 75, 78, 79).

Pronotum slightly convex and distinctly transverse, about twice as wide as long, distinctly broader than head, widest in middle, more narrowed posterad than anterad; apical margin slightly rounded, about as broad as posterior margin, anterior (Fig. 75) and posterior angles (Fig. 80) widely rounded; laterobasal margins slightly concave; lateral margins in middle narrowly marginate; median disc of pronotum with very indistinct transverse and laterobasal portions with indistinct wide impressions (Figs 73–76, 80). Pronotum with more or less regular small and sparse punctation, sometimes with wide impunctate longitudinal area on disc, with distinct and moderately large transverse and diagonal microsculpture (Figs 73–76, 80). Scutellum without visible punctures, with distinct isodiametric microsculpture (Figs 73, 74, 76, 80).

Elytra slightly convex, distinctly longer than broad, twice as long as pronotum, from middle slightly widened apicad, reaching apical margin of abdominal tergite IV, with widely rounded apical angles and straight apical margin truncated at suture (Figs 73–76, 78). Punctation markedly denser and deeper than that on pronotum, smaller on basal and apical margins and near scutellum; microsculpture as that on pronotum (Figs 73, 74, 76, 78).

Abdomen slightly narrower than elytra, with small, moderately sparse punctation and fine indistinct microsculpture.

Male. Apical margin of abdominal tergite VIII rounded. Apical margin of abdominal sternite VIII slightly sinuate.

Female. Details of shapes of apical abdominal segment not visible.

######### Remarks.

The present unique piece of amber contains an interesting and rare aggregation of omaliine specimens which apparently belong to one species. Based on the shape of the body and other structures (antennae, maxillary palpus), features of punctation and microsculpture, etc., the species belongs to Eusphalerini or Omaliini. Tarsi of fore- and middle legs are partly visible in one specimen (Fig. 77); tarsi of this specimen has long and indistinctly dense setae on lateral portions of tarsomeres 1–4 that are common in species of the genus *Eusphalerum*. Based on the shape of the body and other morphological details, and lack of additional morphological data, we have not found similar species among extant representatives of the genus, so we here treat this taxon as *Eusphalerum* sp. 1. We did not observe sexual dimorphism in the shape of apical portions of the elytra, which often occurs in *Eusphalerum*, as was observed for *Eu.kanti* sp. nov. above. Furthermore, the morphology of the aedeagus should be studied, as species of the genus are reliably distinguished by the shapes of the median lobe of the aedeagus and the parameres.

######## Eusphalerum

Taxon classificationAnimaliaColeopteraStaphylinidae

sp. 2.

**Figures 81, 82.**
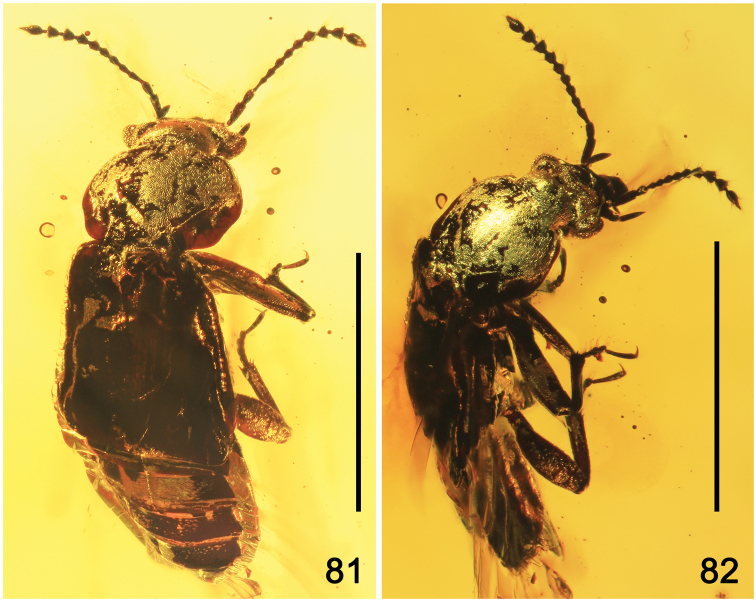
Habitus of *Eusphalerum* sp. 2 **81** oblique dorsal view **82** dorsolateral view. Scale bars: 1.0 mm.

**Figures 83–85.**
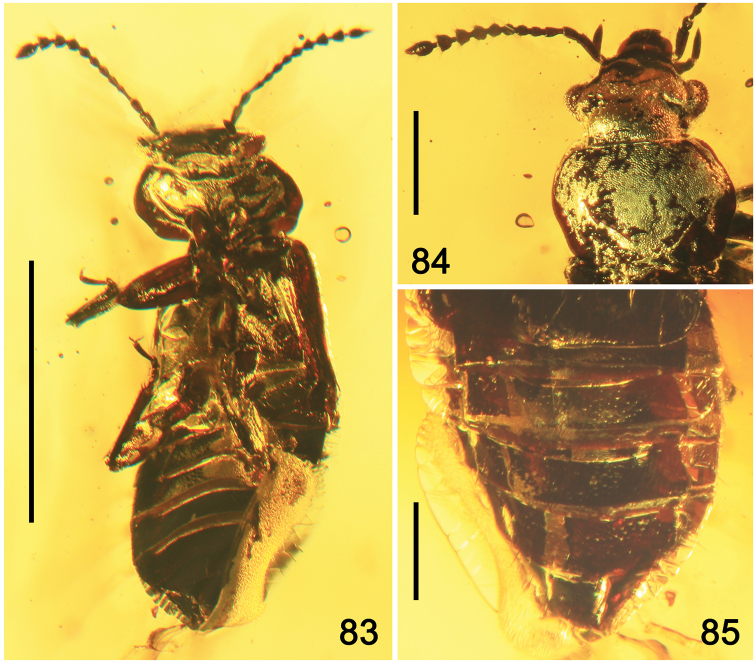
*Eusphalerum* sp. 2 **83** habitus, ventral view **84** left antenna, head and pronotum, dorsal view **85** abdomen, dorsal view. Scale bars: 1.0 mm (**83**), 0.4 mm (**84, 85**).

Figures 12 , 13 , 81–82 , 83–85

######### Materials examined.

One male, complete specimen as an inclusion in a piece of yellow Baltic amber 35.4 mm × 21.5 mm × 7.5 mm in size (Figs 12, 13), with glued very small quadrate paper on plastic bag labeled “AWI | 045”, with the following labels: “AWI-045 | Phyllodrepa (?) | 3 spec.” <rectangular handwritten label>, “Dr. Vitalii Alekseev's | Collection” <rectangular handwritten label>, “*Eusphalerum* sp. 2 | Shavrin A.V. det. 2018” <rectangular label, printed>. The specimen is deposited in the private collection of Vitalii I. Alekseev (Kaliningrad, Russia), registered as AWI-045.

######### Preservation.

The single specimen is a male located close to the outer surface of the piece of amber, with many details visible in both dorsal and ventral surfaces. The elytra are somewhat deformed and seem flattened, and the right elytron is depressed into the thorax. Additionally, the piece of the amber contains two males of *Eu.* sp. 3 and *Eu.* sp. 4 (see below), and a syninclusion located near the narrowest side of the amber: nymph of small mite about 0.50 mm in length (Figs 12, 13).

######### Description.

Measurements: HW: 0.53; HL: 0.20; OL: 0.11; AL: 0.69; PML × PMW (III, IV): III: 0.03 × 0.01, IV: 0.07 × 0.02; PL: 0.43; PW: 0.67; ESL: 0.85; EW: 0.81; MTbL: 0.36; MTrL: 0.27 (I–IV: 0.14; V: 0.13); AW: 0.79; TL: 2.06. Antennomeres with lengths × widths: 1: 0.12 × 0.02; 2: 0.07 × 0.02; 3: 0.06 × 0.02; 4: 0.05 × 0.02; 5–6: 0.05 × 0.03; 7: 0.04 × 0.04; 8: 0.05 × 0.04; 9–10: 0.05 × 0.05; 11: 0.10 × 0.04.

Body elongate, somewhat flattened (Fig. 81), glossy and glabrous, without visible setation. Body appears dark-brown, with basal portions of pronotum and legs reddish-brown. Body dorsolaterally as in Figure 82 and ventrally as in Figure 83.

Head strongly transverse, distinctly more than twice as wide as long, with slightly convex middle portion and posterior parts of infraorbital ridges, without visible grooves in front of ocelli and postocular carina (Fig. 84). Head with indistinct, small and sparse punctation, with moderately coarse and large isodiametric microsculpture between punctures becoming more transverse toward middle part of neck. Eyes large, strongly protruding laterad. Ocelli small, convex, situated very close to infraorbital ridges at level of about middle length of eyes, distance between ocelli about twice as long as distance between ocellus and margin of eye (Figs 82, 84). Labrum transverse. Apical segment of maxillary palp elongate, slightly wider in middle than penultimate segment, from apical third gradually narrowed toward moderately acute apex (Figs 82, 84). Gular sutures not fully visible, widely separated from each other (Fig. 83). Antenna moderately long, distinctly exceeding shoulders of elytra, with elongate setae, distinctly longer on antennomeres 6–11; basal antennomere wide, gradually widened apicad, antennomere 2 similar in width, swollen and elongate, 3 with thin basal portion, slightly widened apicad, 4 slightly shorter than 3, 5, and 6 slightly wider than 4, 7 short and moderately rounded, 8–10 slightly transverse, apical antennomere more than twice as long as broad, from about middle strongly narrowed toward acute apex (Figs 81–84).

Pronotum 1.5 times as wide as long, slightly broader than head, widest in middle, markedly more narrowed posterad than anterad; apical margin slightly and widely rounded, about as broad as posterior margin, anterior and posterior angles widely rounded; laterobasal margins slightly concaved, with very indistinct small crenulation; lateral margins narrowly explanate; lateral portions with indistinct semioval impression about middle (Figs 81, 84). Pronotum with somewhat regular small and sparse punctation and with isodiametric ground sculpture slightly coarser than that on head (Figs 81, 84). Prosternum with moderately wide and protruded prosternal process (Fig. 83). Scutellum large and wide (Fig. 81).

Elytra little longer than wide, about twice as long as pronotum, gradually widened apicad, reaching basal to apical margins of abdominal tergite IV, with widely rounded apicolateral angles; shoulders moderately widely rounded; lateral edges narrowly explanate (Fig. 81). Punctation of elytra invisible in details but appears slightly denser and deeper than that on pronotum.

Legs moderately long and slender, femora markedly widened in middle, tibiae moderately short and thin, gradually widened apicad, slightly shorter than femora, covered by elongate setae, with a few strong setae on apical margins near apex; tarsomeres 1–4 distinctly wide, with dense and long setae; apical metatarsomere long, yet slightly shorter than length of preceding tarsomeres together; tarsal claws simple, elongate (Figs 81–83).

Abdomen (Fig. 85) slightly narrower than elytra; abdominal tergites with sparse and moderately small punctures, no wing-folding patches are visible.

Male. Apical margin of abdominal tergite VIII slightly rounded. Apical margin of abdominal sternite VIII slightly sinuate.

Female unknown.

######### Remarks.

As in the previous species, this specimen has very long and moderately dense setae on lateral portions of tarsomeres 1–4, distinctly deformed body (especially elytra) and unusually strongly protruded eyes.

######## Eusphalerum

Taxon classificationAnimaliaColeopteraStaphylinidae

sp. 3

**Figures 86–90.**
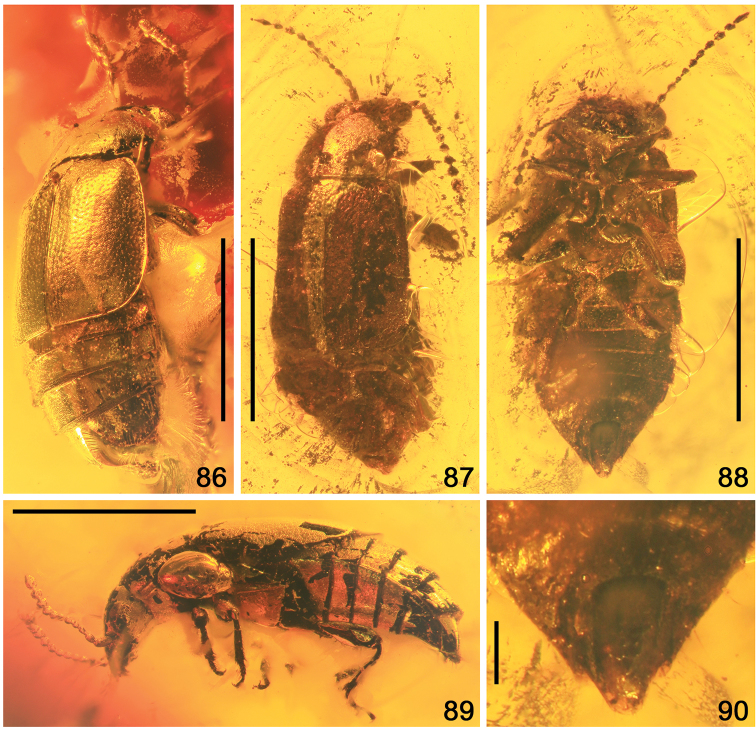
*Eusphalerum* spp. (**86, 89***Eu.* sp. 3; **87, 88, 90***Eu.* sp. 4) **86** habitus, dorsolateral view **87** habitus, oblique dorsal view **88** habitus, ventral view **89** habitus, lateral view **90** abdominal apex, ventral view. Scale bars: 1.0 mm (**86–89**), 0.2 mm (**90**).

Figures 12 , 13 , 86 , 89

######### Materials examined.

One male, an inclusion in the same piece of the Baltic amber that contains a specimen of *Eu.* sp. 2 and *Eu.* sp. 4, with an additional label: “*Eusphalerum* sp. 3 | Shavrin A.V. det. 2018” (private collection of Vitaly Alekseev (Kaliningrad, Russia), registered as AWI-045).

######### Preservation.

The specimen is located dorsolaterally close to the margin of the piece of amber (Figs 12–13). Antennae, lateral potion of the pronotum and elytra with details of the structure of punctation and microsculpture, abdomen and legs (partly) are visible in a dorsal view of the body (Fig. 86); eyes, antennae, some details of thorax, legs and abdomen are relatively visible in a lateral view of the body (Fig. 89).

######### Remarks.

This specimen is about 2.30 mm long (Figs 86, 89). It is similar to *Eu.kanti* sp. nov. and *Eu.* sp. 4 in the shape of the body, eyes and antennomeres. Because some morphological details of head, pronotum, thoracic sclerites, and legs, as well as punctation and microsculpture, are not visible, we leave this specimen unnamed.

######## Eusphalerum

Taxon classificationAnimaliaColeopteraStaphylinidae

sp. 4

Figures 12 , 13 , 87–88 , 90

######### Material examined.

One male, as an inclusion in the same piece of the Baltic amber that contains *Eu.* sp. 2 and *Eu.* sp. 3, with an additional label: “*Eusphalerum* sp. 4 | Shavrin A.V. det. 2018” (private collection of Vitaly Alekseev (Kaliningrad, Russia), registered as AWI-045).

######### Preservation.

The specimen is located with its dorsal side near the widest outer margin of the piece of amber (Figs 12, 13). It is relatively clouded with many details not visible both dorsally (Fig. 87) and ventrally (Fig. 88).

######### Remarks.

This specimen is about 2.30 mm long (Figs 87, 88). Based on the relatively narrow body and shapes of antennomeres, as well as the punctation and microsculpture of the forebody, it is similar to *Eu.* sp. 2. However, we consider this specimen belongs to a different species, because the eyes of this specimen are widely rounded as in *Eu.kanti* sp. nov. and *Eu.* sp. 3, and because some details of the body such as dorsal portion of the head and shapes of front and middle tarsi are poorly visible. Apical part of the abdomen (ventral view) as in Figure 90, with sternite VII distinctly emarginated medioapically.

## Discussion

Here we report on a remarkable, unexpected palaeodiversity of the Omaliinae fauna in Baltic amber. The discovery of five new species and four additional unnamed taxa is noteworthy for several reasons. First of all, the fossil records of Omaliinae in general are fragmentary, resulting in a significant lack of fossil information for this group. So far, omaliines seem to be relatively “prevalent” in the available fossil record but these only reflect a tiny fraction of the complete diversity of Omaliinae. Furthermore, many of these fossils may not be correctly placed systematically, especially for older records. Therefore, there are only few reliable omaliine fossils known so far. Even from Baltic amber, which is one of the most famous and long-studied fossil deposits, only two definitive omaliine species in the tribe Omaliini have been described (Zanetti et al. 2016), with another doubtful taxon considered (Schaufuss 1890). Our discovery of multiple, well-preserved omaliine fossils in Baltic amber adds new insight into the species composition and diversity of Omaliinae during the Eocene. Second, we found several fossils of the genera *Geodromicus* (Anthophagini), *Eusphalerum* (Eusphalerini), *Paraphloeostiba*, and *Phyllodrepa* (Omaliini). Excluding older, doubtfully placed taxa, our fossils of *Eusphalerum* represent the first definitive records of the genus and its monobasic tribe. *Geodromicusbalticus* represents the second and the first definitive fossil record of Anthophagini, with the Eocene *G.abditus* from Florissant, USA, and a probable Jurassic anthophagine from Daohugou, China (Cai and Huang 2013). They extend the origin of each genus at least to the mid-Eocene (ca 44 Ma) and provide several fossil calibration points for future divergence dating of phylogenies. It is highly likely that *Paraphloeostiba* and *Phyllodrepa* had diversified by the mid-Eocene, and our study illuminates the diversity of omaliine assemblages in amber-producing Baltic forests. Third, we describe these fossils using modern photography and used micro-CT imaging for one inclusion. There are only a few examples of micro-CT scanning for staphylinid inclusions in Baltic amber (e.g., Yamamoto and Maruyama 2018; Jałoszyński et al. 2018), although there are additional examples in using PPC-SR X-ray mCT (Zanetti et al. 2016). The techniques applied in this study enable us to systematically place fossil taxa much more precisely and allow for the detailed comparison with other taxa based on concrete evidence. The palaeobiogeogaphy of *Paraphloeostiba* and *Phyllodrepa* was previously discussed based on occurrence of these genera in Baltic amber (Zanetti et al. 2016). Unlike most Omaliinae, *Paraphloeostiba* is a rare example of a thermophilous taxon, while *Phyllodrepa*, in the restricted sense, is more typically temperate loving (Zanetti et al. 2016). Notably, *Paraphloeostibamorosa* sp. nov. is externally similar to *P.specularis*, which is known from the tropical Bismarck Archipelago, Papua New Guinea. It is interesting to understand the reason behind such a puzzling mixture of thermophilous and temperate beetle elements in Baltic amber (Zanetti et al. 2016). The occurrence of *Paraphloeostiba* potentially indicates a higher diversity of thermophilic rove beetles than is currently known (e.g. Brunke et al. 2017) and further supports distinctly warm palaeoclimatic conditions for the Eocene amber forest of Europe. Several examples of palaeodistributions for Baltic amber beetles are discussed by Alekseev (2017). Together with the work of Zanetti et al. (2016), our study forms a foundation for study of the Baltic amber Omaliinae.

A preliminary generic placement of some described fossil species was necessary based on an absence of modern phylogenetic revisions, which would provide clear morphological limits between genera. This applies to the genus *Geodromicus* and other related taxa of the *Hygrogeus* complex, some genera of which were described based on limited morphological characters, such as proportions of the body, shapes of maxillary palpomeres and aedeagus (e.g. Tronquet 1981; Zerche 1992). Some of these characters are distinctly variable and character states overlap. This is also true for *Paraphloeostiba*, which was erected by Steel (1960a) for very diverse groups of Omaliini related to *Phloeostiba* and based on a limited number of characters. Besides *Paraphloeostiba*, several related genera were described from the Oriental Region (e.g. Steel 1959, 1960a, 1960b). However, all of them need revision and a clarification of their limits and phylogenetic relationships based on analysis of both morphological, and molecular data would be extremely desirable. Members of the diverse anthophilous genus *Eusphalerum* and the tribe Eusphalerini are also in need of similar studies and are relevant for higher classification. The morphological data extracted here from extinct species, in some sense, can be useful in the future for understanding boundaries of extant taxa.

Among the extinct species of Omaliinae described here, *G.balticus* sp. nov. raised the most interest as it is the first representative of the tribe Anthophagini recorded in Baltic amber. All the known species of *Geodromicus* are strongly temperate, mostly rheophilous, and inhabit alluvial and other communities connected to rivers, streams, and other water courses. Species of *Geodromicus* and related genera are predators of various small invertebrates, which is reflected in the morphological features of the body, such as elongated legs, antennae, and mouthparts, development and strengthening of teeth on inner margin of each mandible. The newly described species appear to have potentially lived in riparian areas or wet biotopes with mosses and hygrophilous plants, which were distributed in ancient amber-producing forests. Rheophilous and even water beetles are insufficiently known from Baltic amber (see the list of described Coleoptera from the European ambers in Alekseev 2017). Unlike *Geodromicus*, species of the genus *Eusphalerum* are pollen-feeding species, attracted to flowers of various plants (e.g. Zanetti 2014), and very often representatives of this genus aggregate in flowers in huge numbers. The extant species of the other two genera, *Paraphloeostiba* (some species) and *Phyllodrepa* inhabit litter, mosses, decaying plant debris, and sometimes hygrophilous and hygromesophilous communities or nests of birds and mammals (some species of *Phyllodrepa*). It is interesting that some species such as the widespread *Ph.floralis* (Paykull), *Ph.nigra* (Gravenhorst) and some other species are attracted to flowers and known as pollen-feeders (Steel 1970). It can be assumed, hypothetically, that together with *Eusphalerum*, these pollen-feeding species were widely distributed in Cenozoic amber forests, and to the present time are fragmentary preserved in the Holocene fauna with an overwhelming number of species distributed in the mountain regions of the Holarctic Region.

## Supplementary Material

XML Treatment for Geodromicus balticus

XML Treatment for Eusphalerum kanti

XML Treatment for Paraphloeostiba morosa

XML Treatment for Phyllodrepa daedali

XML Treatment for Phyllodrepa icari

XML Treatment for Eusphalerum

XML Treatment for Eusphalerum

XML Treatment for Eusphalerum

XML Treatment for Eusphalerum
